# Controlling and probing non-abelian emergent gauge potentials in spinor Bose-Fermi mixtures

**DOI:** 10.1038/ncomms9135

**Published:** 2015-09-02

**Authors:** Nguyen Thanh Phuc, Gen Tatara, Yuki Kawaguchi, Masahito Ueda

**Affiliations:** 1 RIKEN Center for Emergent Matter Science (CEMS), Wako, Saitama 351-0198, Japan; 2 Department of Applied Physics, University of Tokyo, 7-3-1 Hongo, Bunkyo-ku, Tokyo 113-8656, Japan; 3 Department of Physics, University of Tokyo, 7-3-1 Hongo, Bunkyo-ku, Tokyo 113-0033, Japan

## Abstract

Gauge fields, typified by the electromagnetic field, often appear as emergent phenomena due to geometrical properties of a curved Hilbert subspace, and provide a key mechanism for understanding such exotic phenomena as the anomalous and topological Hall effects. Non-abelian gauge potentials serve as a source of non-singular magnetic monopoles. Here we show that unlike conventional solid materials, the non-abelianness of emergent gauge potentials in spinor Bose-Fermi atomic mixtures can be continuously varied by changing the relative particle-number densities of bosons and fermions. The non-abelian feature is captured by an explicit dependence of the measurable spin current density of fermions in the mixture on the variable coupling constant. Spinor mixtures also provide us with a method to coherently and spontaneously generate a pure spin current without relying on the spin Hall effect. Such a spin current is expected to have potential applications in the new generation of atomtronic devices.

## Abstract (web summary)

Abelian and non-abelian emergent gauge fields govern the physics of many topological phenomena, such as Hall effects. Here the authors show theoretically that in a spinor Bose-Fermi mixture the non-abelianness of the gauge potential can be tuned, offering the chance to study the abelian-non-abelian crossover.

## Graphical abstract

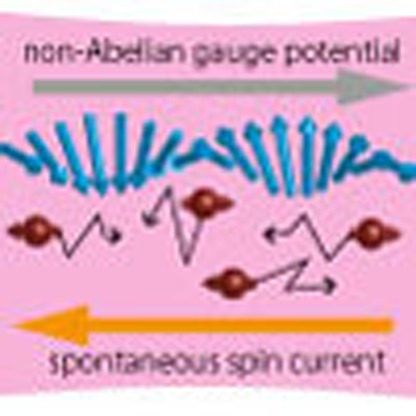

Gauge fields are the fundamental ingredient in the Standard Model of elementary-particle physics^1^. In condensed matter physics, a large number of phenomena including optical and transport properties of solids appear as the responses of the system to an applied electromagnetic field. Remarkably, gauge fields often emerge due to a nontrivial topology of the Hilbert subspace to which the particle's wavefunction is restricted^2^. It is analogous to the parallel transport of a vector on the surface of the two-dimensional unit sphere *S*^2^ as it picks up the surface's curvature to change its direction while going around a closed loop. Here the vector and the *S*^2^ surface corresponds to the wavefunction and the restricted Hilbert subspace, respectively. If a particle with spin degrees of freedom moves in a spatially varying magnetic field and its spin adiabatically follows the field's direction, the particle accumulates a quantum-mechanical phase known as the Berry phase^3^. This phase arises from geometric properties of the Hilbert subspace composed of the spin and motional degrees of freedom, and it serves as an emergent gauge potential in the Hamiltonian description^4^. Since the spin degrees of freedom of the particle are frozen by the adiabatic orientation of its magnetization to the local direction of the external magnetic field, the emergent gauge potential is abelian just like the vector potential of a conventional electromagnetic field. The potential generates forces from its spatial and temporal variations^5^, and these forces act on a spin-carrying particle in the same way as the Lorentz forces act on a charged particle. Examples include the anomalous Hall effect in magnetic materials due to non-coplanar spin configurations^6^,^7^ and the topological Hall effect, which has been observed in both chiral magnets^8^,^9^,^10^ and atomic Bose–Einstein condensates (BECs)^11^ with skyrmion spin textures.

On the other hand, in two-dimensional material systems, almost every possible linear combination of the Rashba^12^ and Dresselhaus^13^ spin–orbit interactions can be represented by a spatially uniform non-abelian vector potential **A**=∑_*α*=*x*,*y*,*z*_ **A**^*α*^*σ*_*α*_, where *σ*_*α*_'s are the Pauli matrices^14^. Due to the non-commutativity [*σ*_*α*_, *σ*_*β*_]≠0, the non-abelian gauge potential generates an effective magnetic field proportional to *σ*_*z*_. This magnetic field points in the opposite directions for spin-up and spin-down particles, thereby giving rise to the spin Hall effect that underlies a large number of spintronics devices^15^,^16^,^17^,^18^,^19^,^20^,^21^. The spin Hall effect has also been extensively studied and recently observed in systems of ultracold atoms with light-induced gauge potentials^22^,^23^,^24^. The idea of a non-abelian gauge potential was first proposed by Wilczek and Zee who generalize the adiabatic theorem to the case in which there are a group of eigenstates that remain degenerate and well isolated from other levels in the course of time evolution^25^. This analysis has led to applications in many areas such as molecular and condensed matter physics^26^,^27^. In particular, the possibility of generating non-abelian magnetic monopoles was proposed and demonstrated in the rotational dynamics of diatomic molecules^28^,^29^,^30^,^31^, nuclear quadrupole resonance^32^ and spinor BECs^33^. The studies of non-abelian dynamics of ultracold atoms in laser fields have been conducted extensively^34^,^35^,^36^,^37^.

So far the abelian and non-abelian gauge potentials have been investigated in different systems, and we have no way to control the non-abelianness of the gauge potentials in a single system. Moreover, there has been no observable indicator of the non-abelianness for partially non-abelian gauge potentials. In this work, we show that a spinor Bose-Fermi mixture can be used to serve this purpose. Such a mixture offers a platform for the study of the abelian–non-abelian crossover, where a change in the singularity of a topological defect such as Dirac's magnetic monopole should play an important role. The mixture we consider consists of an optically trapped ultracold Fermi gas that overlaps with a spinor BEC, which forms a helical spin texture (see Fig. 1). A noncollinear spin texture gives rise to an emergent gauge potential that acts on fermions in the mixture due to their spin-dependent interaction with bosons. We can switch from the abelian to non-abelian regimes of the emergent potential by gradually liberating the spin degrees of freedom of fermions, or equivalently by weakening the spin adiabaticity in the sense of decreasing the ratio of the magnitude of the fermion–boson interaction to the Fermi energy. This can be implemented by varying the relative particle-number densities of bosons and fermions in the mixture. The non-abelian gauge potential then generates a generalized non-abelian electric field, which causes a pure spin current of fermionic atoms to flow in the mixture (see discussions below equation (6)). The non-abelianness of the emergent gauge potential is characterized by an explicit dependence of the spin current density in the Fermi gas on the variable coupling constant. Unlike conventional solid materials, a pure spin current density can be measured in ultracold atomic gases by using the time-of-flight absorption imaging in conjunction with the Stern–Gerlach experiment^38^. The flows of particles of opposite spins in opposite directions would result in two spatially separated expanding clouds of up-spin and down-spin atoms as the trap is removed. Alternatively, the spin accumulations at the edges of the system as a result of the generated spin current can be observed by an *in situ* spin-texture-resolved measurement^39^. Using the non-equilibrium Green's function method, we calculate the spin current density in both strong-coupling and weak-coupling limits and obtain its analytic expression in terms of system's fundamental parameters. Our result is valid over a large parameter region, and thus allows a quantitative comparison between theoretical predictions and experimental measurements. Spinor mixtures also provide us with a new tool to coherently and spontaneously generate a pure spin current, which is not a simple task in solid materials, without relying on the spin Hall effect. Such a spin current is expected to have potential applications in the new generation of ultracold atom-based ‘atomtronic' devices^40^,^41^,^42^,^43^,^44^.

## Results

### System

**Figure 1:**
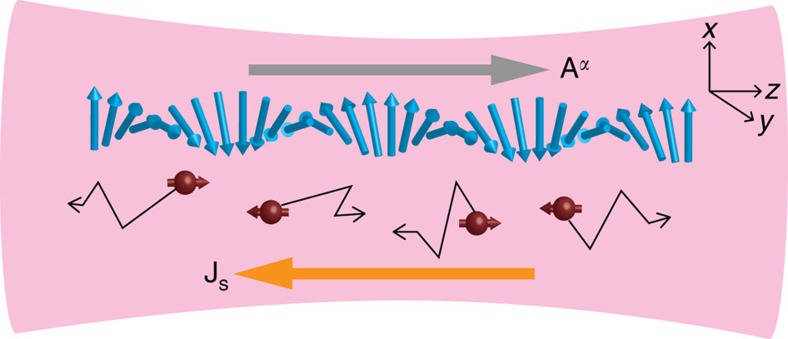
Non-abelian emergent gauge potential in a spinor Bose-Fermi mixture. A Bose-Fermi mixture is confined in an optical trap shown in magenta, where solid blue arrows show how the local magnetization of a ferromagnetic spinor Bose–Einstein condensate varies over space to form a helix, while solid brown spheres represent spin-1/2 fermions with arrows showing their spin's directions. Thin zigzag arrows illustrate diffusive motion of fermions. A helical spin texture is prepared in the condensate through application of a *π*/2 radio-frequency pulse followed by a magnetic field gradient^46^. Fermions experience a non-abelian gauge potential **A**=∑_*α*=*x*, *y*, *x*_ **A**^*α*^*σ*_*α*_ (long grey arrow) with *σ*_*α*_'s being the Pauli matrices due to their interactions with the spin texture. The non-abelianness can be controlled continuously by varying the relative particle-number densities of bosons and fermions. The emergent gauge potential results in a pure spin current **J**_s_ (long orange arrow) in the Fermi gas, that is, flows of fermions with opposite spins in opposite directions, which can be measured using the time-of-flight absorption imaging in conjunction with the Stern–Gerlach experiment.

A spinor Bose-Fermi mixture considered in this work is illustrated in Fig. 1. A helical spin texture is formed in a spinor BEC with a ferromagnetic interaction, such as a spin-1 ^87^Rb condensate, through application of a *π*/2 radio-frequency (RF) pulse to the *z*-axis polarized BEC to rotate the atomic magnetization to the *xy* plane before applying a transient magnetic field gradient d*B*_*z*_/d*z* for a period of *τ*_*B*_ (ref. 45). The Larmor precession of atomic spins with a space-dependent frequency results in a helical spin texture *F*_+_(**r**)≡*F*_*x*_(**r**)+*iF*_*y*_(**r**)=*e*^*i***κ̇r**^ and *F*_*z*_(**r**)=0, where **F**(**r**) represents the unit vector showing the direction of the magnetization of the condensate^46^. The wavevector of the helical spin structure is given by **κ**=(*g*_*F*_*μ*_B_*τ*_*B*_**e**_*z*_/*ℏ*) (d*B*_*z*_/d*z*), where *g*_*F*_ is the Lande *g*-factor for the hyperfine spin-*F* manifold, *μ*_B_ is the Bohr magneton and **e**_*z*_ denotes the unit vector in the *z* direction.

Since the mixture of atomic gases is a dilute system at temperature much lower than the energy scale of the short-range interactions between atoms, the boson–fermion interactions can be well approximated by contact interactions whose strengths are determined by the *s*-wave scattering lengths: 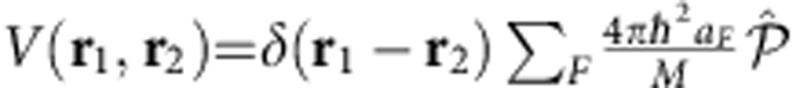, where 
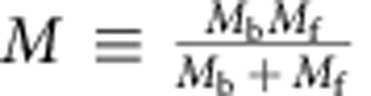
 is the effective mass for the relative motion of a boson with mass *M*_b_ and a fermion with mass *M*_f_, and *a*_*F*_ and 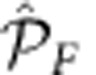 are the scattering length and the projection operator onto the total hyperfine spin-*F *scattering channel, respectively. For the scattering of a spin-*S* boson (*S* is an integer) and a spin-1/2 fermion, *F* can take one of the two values 
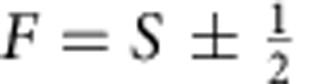
. On the other hand, the projection operators can be linearly expressed in terms of the identity operator and the spin product operator as 
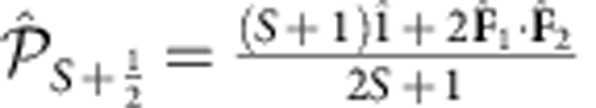
 and 
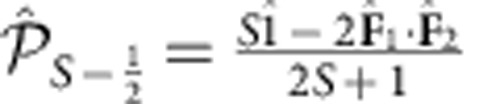
; thereby the interaction can be rewritten as


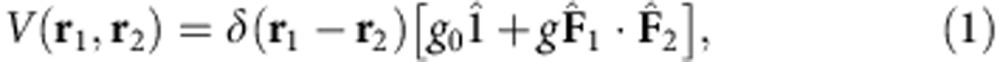


where 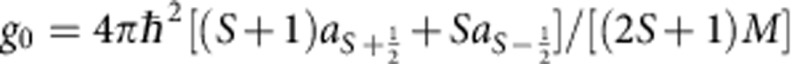 and 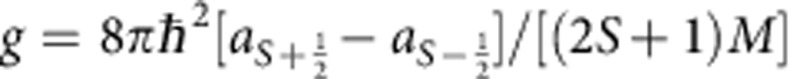 are the spin-independent and spin-dependent interaction strengths, respectively. In the second-quantization representation, the spin-dependent interaction between bosons and fermions is given by


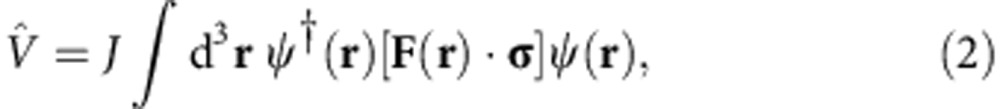


where 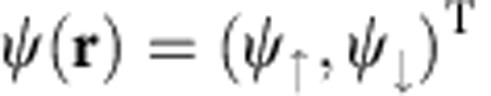 is the annihilation operator of the fermions (T denotes the transpose), **σ** is the vector of Pauli matrices and *J*=*gSn*_b_/2 is the coupling constant with *n*_b_ being the density of condensate particles. Here we used the fact that the BEC has a macroscopic occupation of atoms in a single-particle state so that their field operators can be approximated by a classical field with the magnetization vector given by **F**(**r**). In the following calculations, the spin texture is assumed to be time independent. The validity of this assumption and the roles of other interactions will be discussed in the Discussion section.

### Emergent gauge potential

To find the emergent gauge potential, we go to the adiabatic reference frame in which the quantization axis of the spins of fermions is aligned parallel to the local spin texture of the BEC. Mathematically, it is carried out by means of a 2 × 2 unitary matrix *U*(**r**) such that *U*^†^(**r**)[**F**(**r**)·**σ**]*U*(**r**)=*σ*_*z*_. If the unit vector **F**(**r**) is represented by the polar angles as **F**(**r**)=(sin *θ* cos*φ*, sin*θ* sin*φ*, cos*θ*)^T^, *U*(**r**) can be expressed in terms of the Pauli matrices as *U*(**r**)=**m**(**r**)·**σ** with 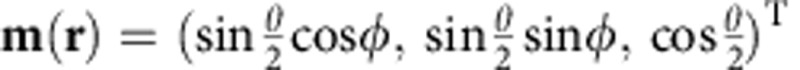. Note that *U*^†^=*U* and *U*^2^=1, implying the unitarity. The field operator 
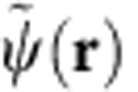
 of fermions in the adiabatic frame is related to its counterpart in the laboratory frame by 
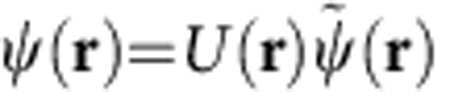
. The Hamiltonian of the Fermi gas 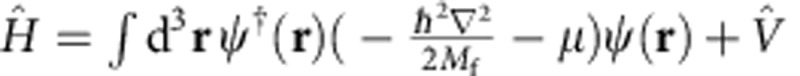, where *μ* is the temperature-dependent chemical potential for a given particle-number density and 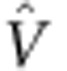 is the interaction given by equation (2), is then rewritten in terms of 
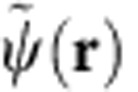
 as^47^


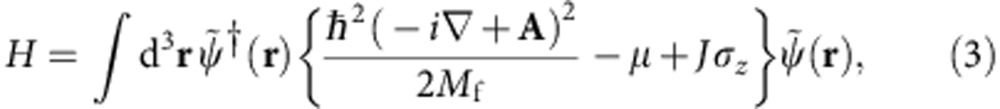


where each component *A*_*j*_ (*j*=*x*, *y*, *z*) of the generalized non-abelian gauge potential is a 2 × 2 matrix given by 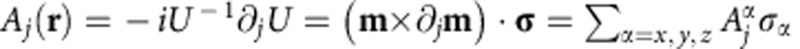. Here and henceforth, the superscripts in greek characters represent the spin polarization, while the subscripts in roman characters denote the spatial direction. In terms of the polar angles *θ* and *φ* of **F**(**r**), the three spin polarization components of the emergent gauge potential are written as:


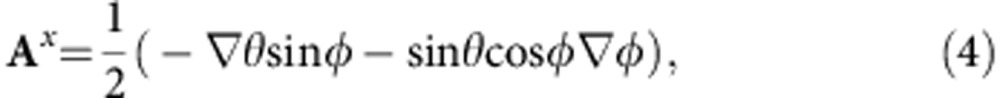



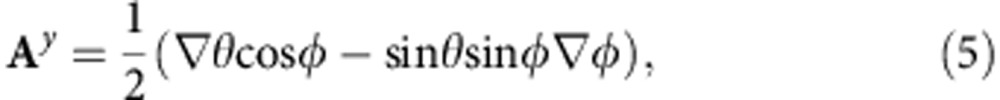



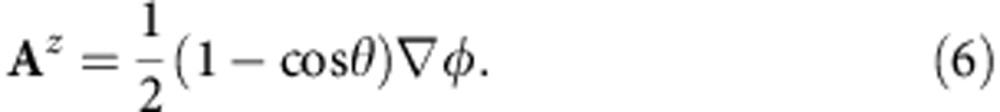


For the helical spin texture with *θ*(**r**)=*π*/2 and *φ*(**r**)=**κ**·**r**, we have **A**^*x*^(**r**)=−**κ** cos(**κ**·**r**)/2, **A**^*y*^(**r**)=−**κ** sin(**κ**·**r**)/2, and **A**^*z*^(**r**) =**κ**/2. The last term on the right-hand side of equation (3), which arises from the interaction (equation (2)) after the unitary transformation, is equivalent to a magnetic Zeeman energy in the adiabatic frame. It is the non-abelian feature of the gauge potential in combination with the Zeeman interaction that generates a generalized non-abelian electric field, which in turn causes a pure spin current to flow in the Fermi gas. This can be seen from the Heisenberg equation of motion for a spin-1/2 particle under the Hamiltonian (equation (3)). Using the algebra of operators, we obtain 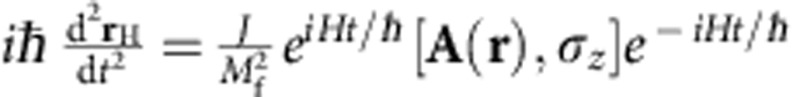, where the subscript H indicates operators in the Heisenberg picture, the [,] denotes the commutator of two operators, and **A**(**r**)=**A**^*x*^(**r**)*σ*_*x*_+**A**^*y*^(**r**)*σ*_*y*_+**A**^*z*^(**r**)*σ*_*z*_ (see Supplementary Note 1 for more details). Due to the non-abelianness in the gauge potential **A**(**r**), the right-hand side of the equation of motion, which depends only on **r** and **σ**, is non-zero, and it can be interpreted as a force exerted on the particle by a generalized non-abelian electric field. It can also be seen from equation (3) that the emergent gauge potential **A**(**r**) is coupled with the spin current density of fermions by 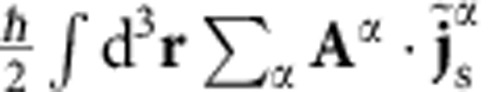, where the spin current density in the adiabatic frame is given by 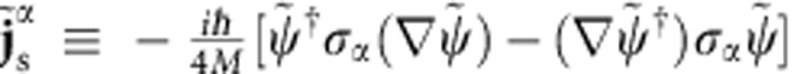. As will be shown in the next section, it is the generalized non-abelian electric field generated by the non-abelian gauge potential that causes a pure spin current of fermionic atoms in the mixture, and the non-abelianness can be characterized by the dependence of the measurable spin current density on the variable coupling constant.

### Spin current density

Using the unitary transformation *U*(**r**) introduced in the previous section, the spin current density operator for spin-1/2 fermions in the laboratory frame 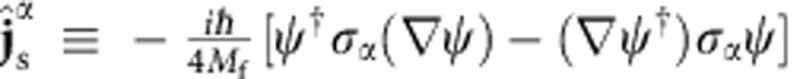 is expressed in terms of the field operators in the adiabatic frame as 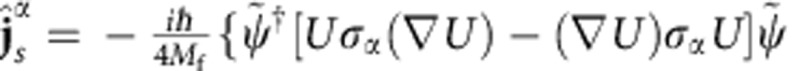

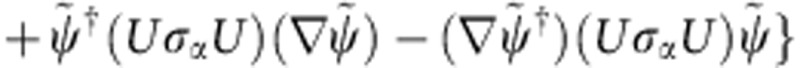
. With *U*(**r**)=**m**(**r**)·**σ**, we obtain the *j* (current's direction) and *α* (spin polarization) component of the spin current density as


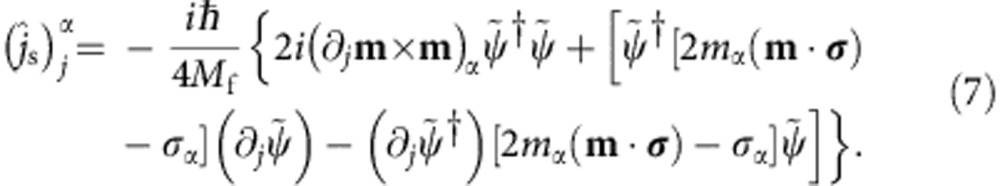


Since
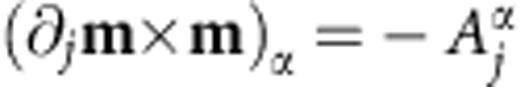
and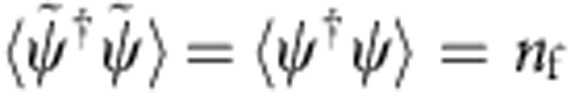 is the particle-number density of fermions, the first term on the right-hand side of equation (7) reduces to


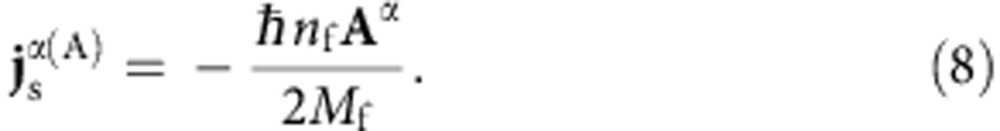


This is the abelian contribution of the emergent gauge potential to the spin current density since it is equal to the value of 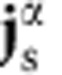 at the limit of |*J*|→∞, at which the spin direction of fermions is always aligned parallel to the local magnetization of the spin texture and thus the spin degrees of freedom are frozen. It is also evident from equation (8) that the different spin polarization components **A**^*α*^ (*α*=*x*, *y*, *z*) of the gauge potential do not mix with each other as opposed to the non-abelian contribution. For the helical spin texture, we have 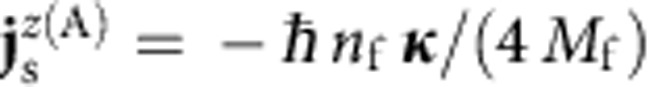. This type of spin current induced by a helical spin texture is related to the spin current mechanism of the electric polarization underlying multiferroic materials^48^,^49^,^50^,^51^. There is also a similarity between this abelian component and the spin current in a fermionic system with the one-dimensional spin–orbit interaction, that is, with equal weights of the Rashba and Dresselhaus components. In both cases, the spin-locking effect, which arises from either the adiabatic alignment of magnetization to the helical spin texture or the spin–orbit interaction, plays the key role in generating a pure spin current.

The remaining terms on the right-hand side of equation (7) give the non-abelian contribution 
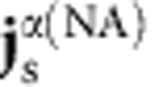
 of the emergent gauge potential to the spin current density. Using the explicit expression of 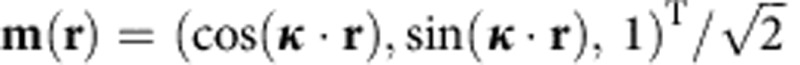 for the helical spin texture, we obtain the spin current density that is polarized in the *z* direction:


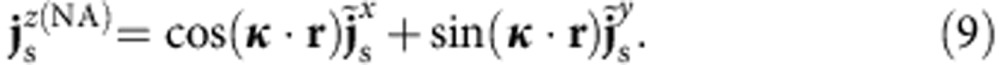


As shown below, 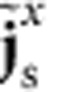 and 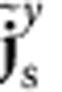, which emerge purely from a non-abelian gauge potential, are non-vanishing for finite *J*, and the mixing of different spin polarization components due to the non-commutativity [*σ*_*x*_, *σ*_*y*_]=2*iσ*_*z*_ characterizes the non-abelian component.

**Figure 2:**
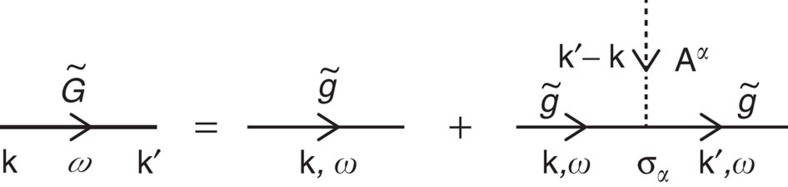
Feynman diagram of a fermion interacting with an emergent gauge potential up to the linear order. The non-abelian gauge potential **A**^*α*^ with *α*=*x*, *y*, *z* denoting the direction of the spin polarization is coupled to the spin current density of fermions in the adiabatic frame, leading to the Dyson equation for the Green's function (equation (10)). The thick, thin and dotted lines represent the interacting (
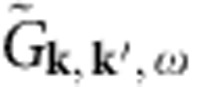
) and non-interacting (
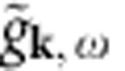
) Green's functions, and the gauge potential, respectively, where 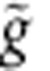 is diagonal in both wavevector **k** and frequency ω. The Pauli matrix *σ*_*α*_ appears due to the spin-dependent coupling of fermions to the emergent gauge potential.

To evaluate 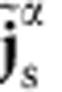 (*α*=*x*, *y*), we use the non-equilibrium Green's function method in which the spin current density can be expressed in terms of the lesser Green's function (see the Methods section). In the adiabatic limit, where either the spin texture varies smoothly in space or the coupling between fermions and the spin texture is strong, we can make a perturbative expansion of the Green's function with respect to the emergent gauge potential **A**^*α*^. This amounts to an expansion in powers of the dimensionless adiabatic parameter 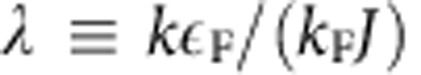, where *k*_F_ and 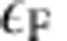 denote the Fermi wavevector and Fermi energy, respectively^52^. Up to the linear order in **A**^*α*^, the Fourier transform of the lesser Green's function is given by


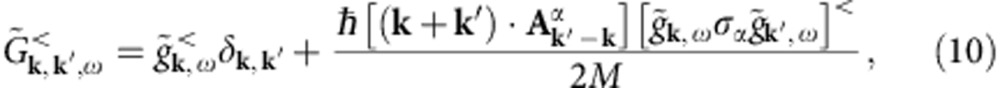


where the superscript < denotes the lesser component of the non-equilibrium Green's function and the summation over *α*=*x*, *y*, *z* was taken. Here 
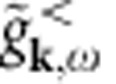
 is the non-interacting Green's function in the adiabatic frame which is diagonal in both the wavevector and the frequency, and 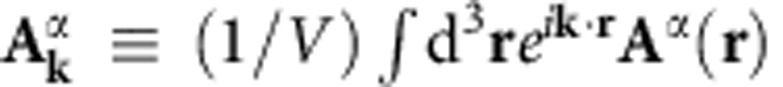 with *V* being the volume of the system. Equation (10) is illustrated by the Feynman diagram in Fig. 2. A straightforward calculation of the non-abelian component of the spin current density yields (see Supplementary Note 2 for the derivation)


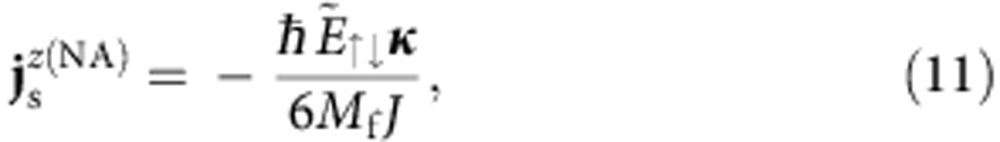


where 
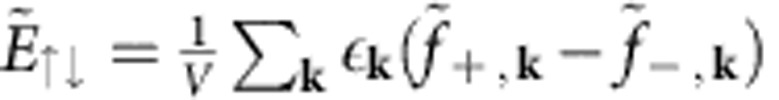
 is the difference in total kinetic energy density between fermions with spin up and those with spin down in the adiabatic frame due to the Zeeman energy (the last term in equation (3)). Here 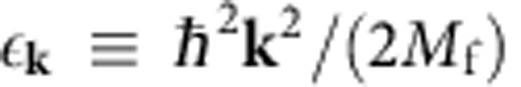, and 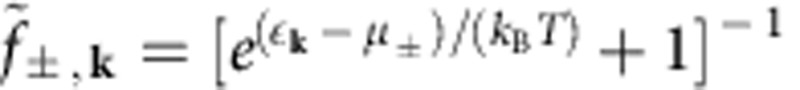 are the Fermi-Dirac distributions with *μ*_±_≡*μJ*. Therefore, the total spin current density is


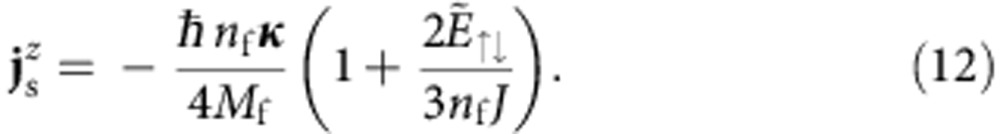


In the strong-coupling limit 
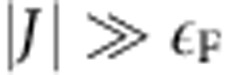
 and at low temperature 
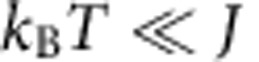
, the difference in kinetic energy density between particles with opposite spins can be obtained analytically as 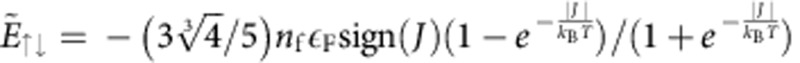. The spin current density then reduces to


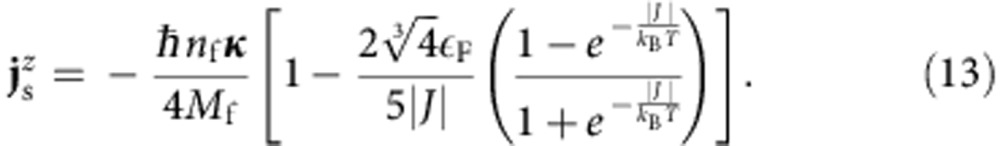


In the opposite weak-coupling limit 
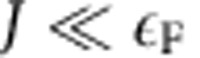
, where the perturbative expansion with respect to **A**^*α*^ does not converge if 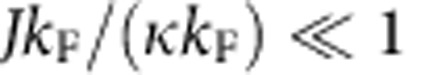, we can make an alternative expansion of the spin current density in powers of 
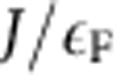
 in the laboratory frame. Using a similar non-equilibrium Green's function approach, the total spin current density is given in terms of the non-interacting Green's function *g*_**k**,*ω*_ up to the second order in *J* as


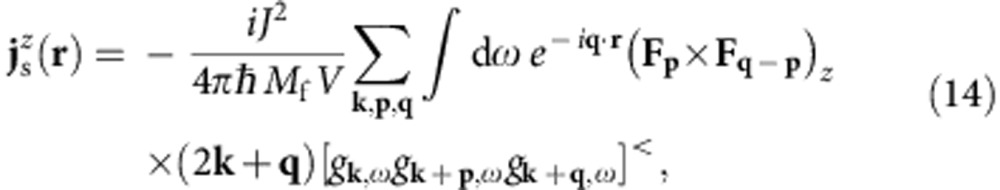


where **F**_**q**_≡(1/*V*)∫d^3^**r** *e*^*i***q**·**r**^**F**(**r**). The cross product of two magnetization vectors on the right-hand side of equation (14) implies that the spin current emerges from a noncollinear spin texture. This factor arises from Tr{*σ*_*z*_[**F**_1_·**σ**][**F**_2_·**σ**]}=2*i*[**F**_1_ × **F**_2_]_*z*_, where **F**·**σ** is the interaction between fermions and the spin texture (equation (2)). For the helical spin texture, we have 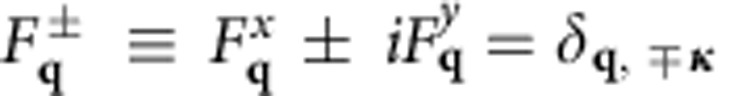. The lesser non-interacting Green's function in the laboratory frame is given by 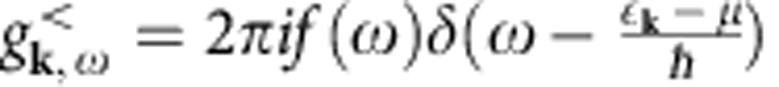, where 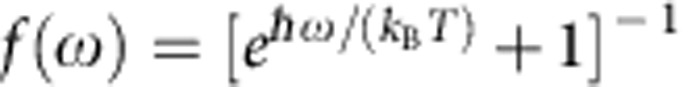. Substituting these in the right-hand side of equation (14) and with a straightforward calculation, we obtain the *z*-axis spin polarization component 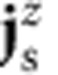 at low temperature 
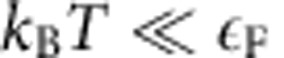
 as (see Supplementary Note 3 for details)


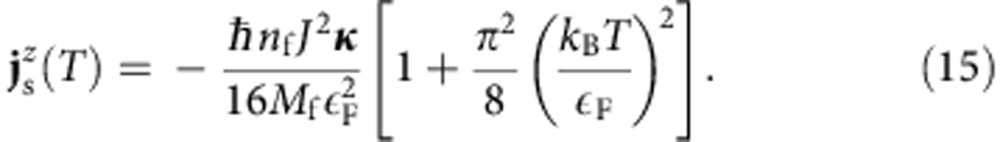


**Figure 3:**
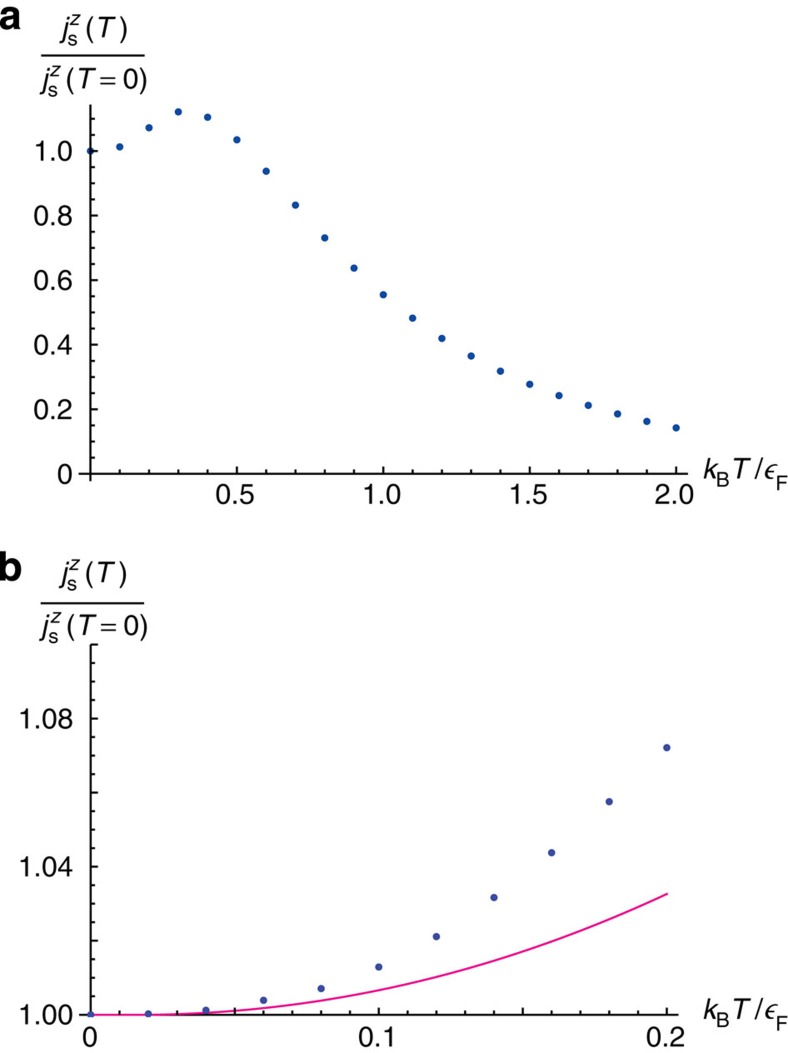
Temperature dependence of the spin current density in the weak-coupling limit. (**a**) Spin current density 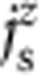 (blue filled circles) normalized by its value at zero temperature is plotted against the dimensionless temperature 
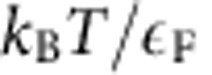
, where *k*_B_ and 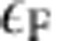 are the Boltzmann constant and the Fermi energy, respectively. (**b**) Blowup of 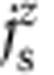 for small 
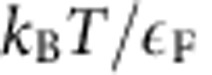
 with the asymptotic behaviour at low temperature (the right-hand side of equation (16)) shown by the curve in magenta for comparison. The initial growth of 
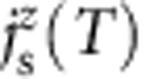
 is attributed to the increasing number of particles with higher velocities, while at higher temperatures the thermal random motion of particles in a non-degenerate Fermi gas and a decrease in the number of condensate particles suppress the directional flow of the spin current. The maximum value of 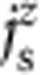 is attained at 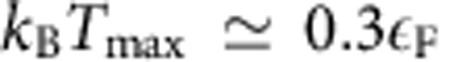.

The other spin polarization components **j**_s_^*x*^ and **j**_s_^*y*^ vanish. It should be noted, however, that the coupling constant *J* is also a function of temperature via the density of condensate particles 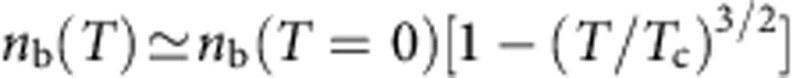, where 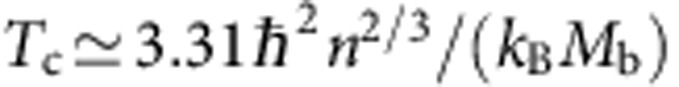 is the critical temperature of Bose–Einstein condensation with *n* being the total particle-number density of bosons. Here we use the model of free bosons, which is a good approximation in evaluating thermodynamic properties of a weakly interacting Bose gas^53^. Therefore, we have


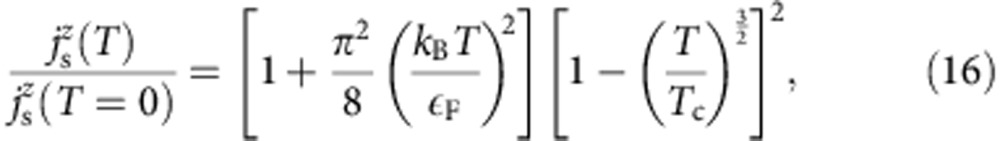


where . Using the same parameters as in ultracold atomic experiments (see the Discussion section below), the critical temperature is estimated to be 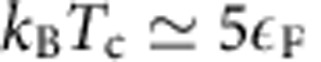. The temperature dependence of the spin current density at higher temperatures is evaluated numerically and the obtained result is shown in Fig. 3. The initial growth of 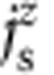 with increasing temperature originates from the broadening of the Fermi-Dirac distribution towards the states with high velocities due to thermal excitations. In contrast, at higher temperatures where the Fermi gas becomes non-degenerate, the thermal random motion of fermions and a decrease in the number of condensate particles suppress the directional flow of the spin current. The maximum value of the spin current density is attained at the temperature given by 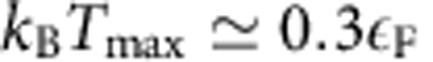.

### Abelian–non-abelian crossover

**Figure 4:**
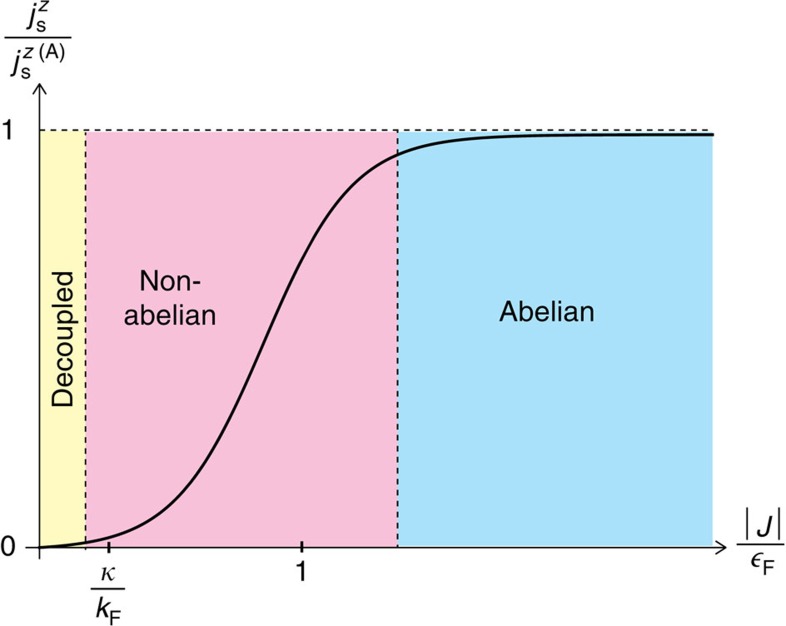
Classification of the emergent gauge potential into different regimes depending on the variable coupling strength in a spinor Bose-Fermi mixture. The behaviour of the magnitude of the *z*-axis spin polarization component 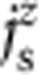 of the spin current density (normalized by its abelian component 
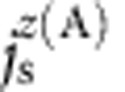
) as a function of the coupling constant *J* (measured relative to the Fermi energy 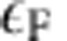) is plotted according to equations (12) and (15). The abelian (blue), non-abelian (magenta) and decoupled (yellow) regimes are identified by 
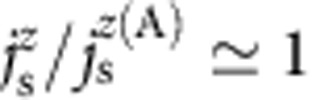
, 
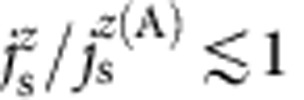
, and 
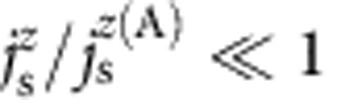
, respectively. The abelian–non-abelian crossover occurs at 
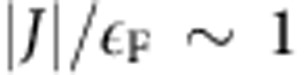
, while for 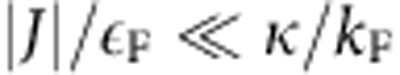, where typically 
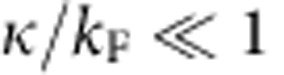
, the interaction between fermions and the spin texture in the Bose–Einstein condensate is so weak that they are essentially decoupled from each other. Since *j*_s_^*z*(A)^ is a constant with respect to the coupling constant, the *J*-dependence of 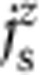 can be used as an evidence of the non-abelian feature of the emergent gauge potential.

From the result obtained in the previous section (equation (12)), the ratio between the non-abelian and abelian components of the spin current density is given by 
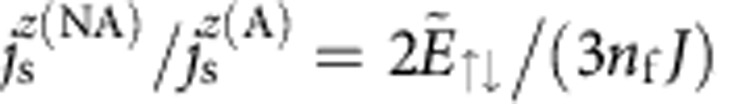
, which is equal to 
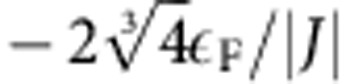
 for 
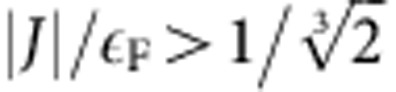
 and *T*=0. This implies that the abelian–non-abelian crossover should occur at 
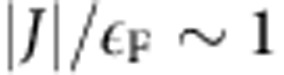
. The investigation of this crossover should be of importance because it relates to the question of how the singularity of a topological defect such as the Dirac magnetic monopole changes as it transforms into a non-singular non-abelian monopole. In contrast, if the coupling constant is so weak that 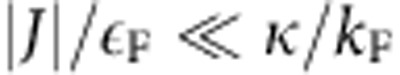, where typically 
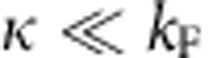
, the fermions are essentially decoupled from the spin texture in the BEC. The classification of the emergent gauge potential into the abelian (
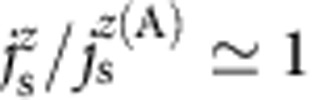
), non-abelian (
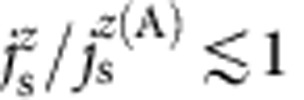
), and decoupled regimes (
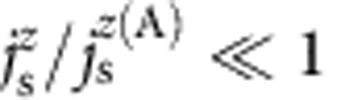
) is schematically shown in Fig. 4, where these different regimes can be identified from the magnitude of the total spin current density relative to its abelian component based on equations (12) and (15).

## Discussion

We now discuss possible experimental situations of a spinor Bose-Fermi mixture in which the non-abelianness of the emergent gauge potential can be controlled, and, consequently, the abelian–non-abelian crossover can be investigated. As shown in Fig. 4, we can move from the abelian to non-abelian regimes by changing the ratio of the magnitude of the coupling constant |*J*| to the Fermi energy 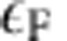. It is given by


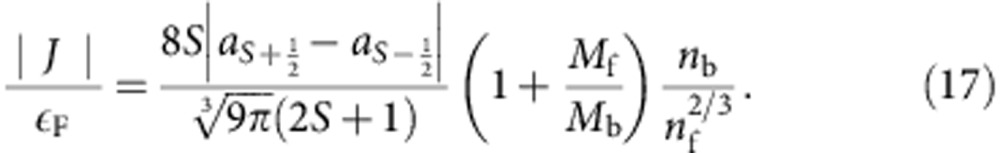


Therefore, the non-abelianness of the emergent gauge potential can be continuously changed by varying the particle-number density of condensed bosons *n*_b_ against fermions *n*_f_. Since the abelian component of the spin current density 
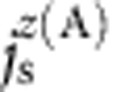
 is a constant if *n*_f_ is held fixed, the *J* dependence of the measurable total spin current density 
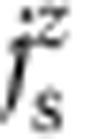
 (with *n*_b_ being varied) will characterize the non-abelian feature of the gauge potential. Alternatively, if *n*_f_ is varied to linearly change the coupling constant *J*, the deviation of the measured 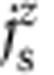 from a linear function of *n*_f_ provides a telltale signal of the non-abelian gauge potential.

To give a concrete example, we consider a mixture of the spin-1 ^87^Rb BEC and the ^6^Li Fermi gas with spin-1/2 (refs 54, 55, 56, 57). Other choices of atomic species for the spinor Bose-Fermi mixture are also possible^58^. To give an estimate of the difference *a*_3/2_−*a*_1/2_ in the *s*-wave scattering length between the two possible total hyperfine spin-*F* channels (*F*=1/2 and 3/2), we have used the numerically calculated atomic potentials^59^ and the corresponding values of the scattering lengths^60^ for the electronic spin-singlet and spin-triplet scattering channels, and calculated the Clebsch–Gordan coefficients for the transformation between the hyperfine- and electronic-spin bases (see the Supplementary Note 4 for details). Here we take |*a*_3/2_−*a*_1/2_|=50 *a*_B_, where *a*_B_ is the Bohr radius. We take the particle-number density of condensed bosons *n*_b_=10^14^ cm^−3^ and that of fermions *n*_f_=10^11^ cm^−3^. Substituting these parameters in equation (17), we obtain 
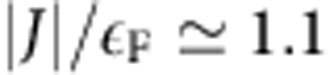
. The ratio between the non-abelian and abelian components of the spin current density is found to be 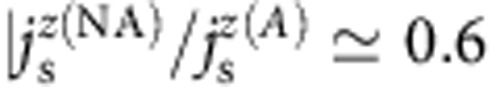. The pitch of the helical spin texture can be adjusted by controlling the duration of the applied magnetic field gradient, and here we take *κ*/*k*_F_=0.1. The magnitude of the spin transport velocity for spin-1/2 particles 
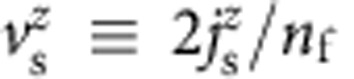
 is found to be about 9.4 × 10^−4^ m s^−1^, implying that a particle with a specific spin polarization propagates through a 100-μm-long atomic cloud in ∼0.1 s. In other words, one-half of particles in the Fermi gas with spin up moves with that velocity in one direction and the other half of particles with spin down moves with the same velocity but in the opposite direction. This kind of spin transport can be measured in ultracold atomic systems using time-of-flight absorption imaging in conjunction with the Stern–Gerlach experiment.

**Figure 5:**
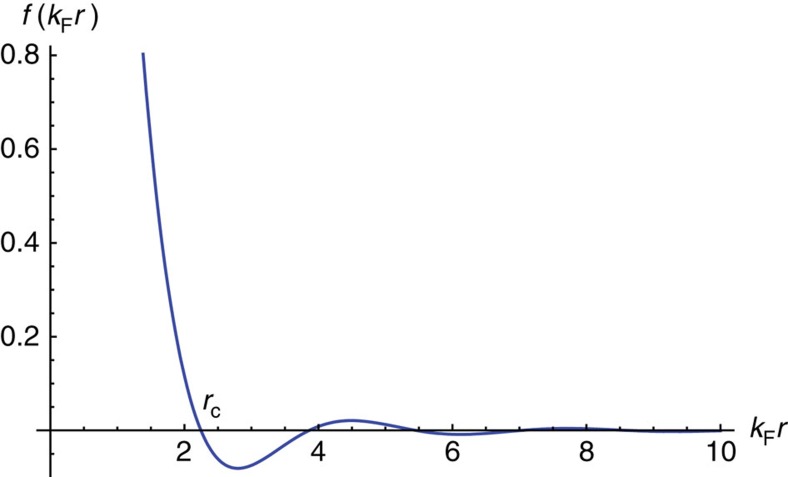
Dependence of the RKKY interaction between two magnetizations on their distance. The RKKY interaction between two magnetizations **F**_1_ at **r**_1_ and **F**_2_ at **r**_2_ that couple to a common three-dimensional Fermi gas is proportional to **F**_1_·**F**_2_ with a coefficient that depends on the distance **r**=**r**_1_−**r**_2_ through the function *f*(*k*_F_*r*) in equation (18). The interaction is ferromagnetic at small distances *r*≤*r*_c_=2.25 *k*_F_^−1^ but its sign oscillates between positive and negative in addition to the rapid damping of its magnitude at large distances.

In this work, we have concentrated on the spin-dependent interaction between bosons and fermions. The effects of the other interactions in the system can be neglected because the spin-independent interaction does not affect the spin transport under consideration. The interaction between fermions, which is typically small in an atomic gas compared with the Fermi energy, should give a negligible effect on the spin current density. However, the helical spin texture in the BEC tends to decay towards a modulated spin structure. There are two mechanisms for this decay. The first is due to the reflection of the supercurrent at the edges of the finite-size BEC as shown by a numerical simulation^61^. The second is due to the backaction of the Fermi gas on the spin texture in the condensate. The backaction can be understood in terms of the RKKY interaction, which is an indirect interaction between two spins mediated by the Fermi sea. Similar to the case of ferromagnetic metals where two localized spins can interact with each other via conducting electrons, the RKKY interaction between two magnetizations **F**_1_ at **r**_1_ and **F**_2_ at **r**_2_ due to a three-dimensional Fermi gas has the form of


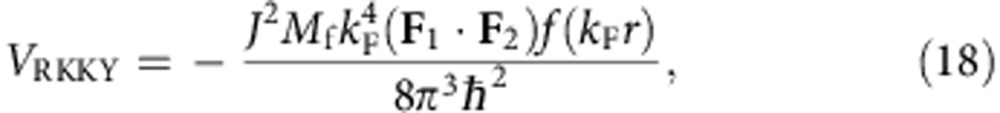


where *r*=|**r**_1_−**r**_2_| and *f*(*x*)≡[sin(2*x*)−2*x* cos(2*x*)]/*x*^4^ (refs 62, 63, 64). As shown in Fig. 5, the RKKY interaction is ferromagnetic at small distances *r*≤*r*_c_=2.25 *k*_F_^−1^ but its sign will oscillate between positive and negative in addition to the rapid damping of its magnitude at large distances. Both of the two mechanisms, however, can be suppressed by loading bosons to an optical lattice with the lattice constant much larger than *k*_F_^−1^ and the lattice depth adjusted by the laser intensity so that each of the subcondensates on the lattice sites has a dimension of the same order or smaller than *r*_c_. The bosonic part of the mixture then consists of an array of almost independent and spin-homogeneous subcondensates whose magnetization's direction varies from one subcondensate to another to form a helical spin texture. This would suppress the supercurrent in the system, while the RKKY interaction between atoms in a single subcondensate has a ferromagnetic nature that helps stabilize the spin configuration. It should be noted that the magnetic dipole–dipole interaction of ^87^Rb is so weak that it gives a negligible effect on the spin structure in a lattice.

To conclude, we have demonstrated that spinor Bose-Fermi mixtures offer an excellent playground for the study of the abelian–non-abelian crossover of the emergent gauge potential as the non-abelianness can be continuously varied. The change in the singularity of a topological defect such as Dirac's magnetic monopole at this crossover deserves further investigation. We can move from the abelian to non-abelian regimes by varying the relative particle-number densities of condensed bosons and fermions in the mixture. The non-abelian feature of the emergent gauge potential is characterized by the dependence of the measurable spin current density on the variable coupling strength. The result of this study also suggests a method to coherently and spontaneously generate a pure spin current without relying on the spin Hall effect and the spin–orbit interaction unlike in conventional solid materials. It is expected that the non-abelian emergent gauge potential will have potential applications in the new generation of ultracold atom-based spintronics, that is, ‘atomtronics' devices.

## Methods

### Non-equilibrium Green's function method

The spin current density 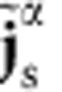 (*α*=*x*, *y*, *z*) in the adiabatic frame can be expressed in terms of the lesser Green's function 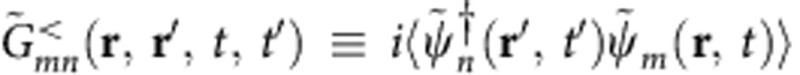 (*m*, *n*=↑, ↓) as


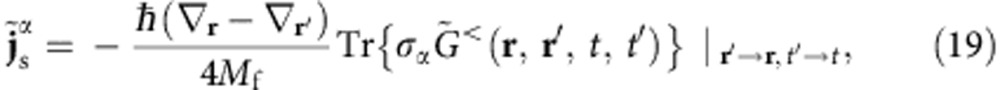


where the trace is taken over the spin indices and ∇_**r**_ indicates the gradient with respect to **r**. On the other hand, the lesser Green's function is related to the Keldysh non-equilibrium Green's function 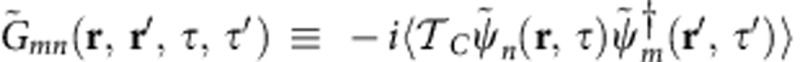 by taking the two arguments on the forward (*τ*∈*C*_←_) and backward (*τ*′∈*C*_→_) parts of the Keldysh contour *C*^65^. Here 
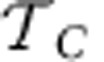
 denotes the path-ordering operator on *C*. Similar to the ordinary Green's function with the time-ordering operator 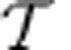 defined on the real-time axis, the Keldysh Green's function satisfies the Dyson's equation. Making a perturbative expansion of the Dyson's equation with respect to the emergent gauge potential, we obtain the Fourier transform 
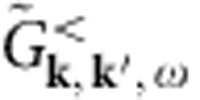
 of the lesser Green's function up to the linear order in **A**^*α*^ as given by equation (10). Here for a time-independent system under consideration, the Green's function 
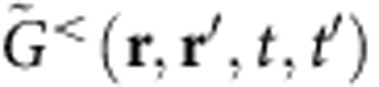
 depends only on the time difference *t*−*t*′; thereby, its Fourier transform depends only on a single frequency *ω*. Substituting equation (10) in equation (9) and further in equation (9) and after a straightforward calculation, we obtain the non-abelian contribution of the emergent gauge potential to spin current density 
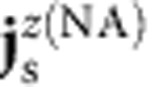
 as given by equation (11). The details of the derivation are presented in Supplementary Note 2.

## Additional information

**How to cite this article**: Phuc, N. T. *et al*. Controlling and probing non-abelian emergent gauge potentials in spinor Bose-Fermi mixtures. *Nat. Commun.* 6:8135 doi: 10.1038/ncomms9135 (2015).

## Supplementary Material

Supplementary Information Supplementary Note 1-4 and Supplementary References
