# Supplementary material for: Controlling and probing non-abelian emergent gauge potentials in spinor Bose-Fermi mixtures
Source: Nat Commun. 2015 Sep 2;6:8135. doi: 10.1038/ncomms9135 (PMC4569724; doi:10.1038/ncomms9135)
Supplement: Supplementary Information — Supplementary Note 1-4 and Supplementary References [file ncomms9135-s1.pdf]

SUPPLEMENTARY NOTE 1: EQUATION OF MOTION OF A SINGLE PARTICLE IN THE PRESENCE OF A NON-ABELIAN GAUGE POTENTIAL

In this section, we derive the equation of motion of a spin-1/2 single particle with mass M in the presence of a non-Abelian gauge potential and a Zeeman interaction. The Hamiltonian of the system in the coordinate-spin composite Hilbert space is given by

$$\begin{aligned} H =& \frac{[\hat{\mathbf{p}}\check{1} - \check{\mathbf{A}}(\hat{\mathbf{r}})]^2}{2M} - J\hat{1}\check{\sigma}_z \\ \equiv& H_1 + H_2, \end{aligned} \tag{1}$$

where $\hat{\mathbf{p}}$ and $\hat{\mathbf{r}}$ are the momentum and coordinate operators, respectively; $\check{\mathbf{A}}(\hat{\mathbf{r}})$ is the non-Abelian gauge potential, where each component of the vector is a linear combination of Pauli matrices $\check{\sigma}_x$, $\check{\sigma}_y$ and $\check{\sigma}_z$; and J is the coupling constant of the Zeeman interaction. Here we use the caret and check symbols to indicate operators in the coordinate and spin spaces, respectively.

We now move to the Heisenberg picture to derive the equation of motion for the operator $\mathbf{r}(t) \equiv e^{iHt/\hbar}\mathbf{r}(t = 0)e^{-iHt/\hbar}$, which is generally an operator in the composite Hilbert space. At $t = 0$, it is given by the tensor product $\mathbf{r}(t = 0) = \hat{\mathbf{r}}\check{1}$ and commutes with the Zeeman interaction term in the Hamiltonian (1). Therefore, $\mathbf{r}(t)$ commutes with the Zeeman term at any time t, and we only need to calculate the commutator between $\mathbf{r}(t)$ and the first term in Eq. (1) to obtain the Heisenberg equation of motion:

$$\frac{\mathrm{d}\mathbf{r}}{\mathrm{d}t} =\frac{1}{i\hbar}[\mathbf{r}(t), H] = \frac{\mathbf{p}(t) - \mathbf{A}(t)}{M} \equiv \frac{\mathbf{\Pi}(t)}{M}, \tag{2}$$

where $\mathbf{p}(t) \equiv e^{iHt/\hbar}\mathbf{p}(t = 0)e^{-iHt/\hbar}$, $\mathbf{A}(t) \equiv e^{iHt/\hbar}\mathbf{A}(t = 0)e^{-iHt/\hbar}$, $\mathbf{p}(t = 0) = \hat{\mathbf{p}}\check{1}$, and $\mathbf{A}(t = 0) = \check{\mathbf{A}}(\hat{\mathbf{r}})$.

Similarly, the Heisenberg equation of motion for $\mathbf{\Pi}(t)$ is given by

$$M\frac{\mathrm{d}^2\mathbf{r}}{\mathrm{d}t^2} = \frac{\mathrm{d}\mathbf{\Pi}}{\mathrm{d}t} =\frac{1}{i\hbar}[\mathbf{\Pi}(t), H]. \tag{3}$$

To evaluate the commutator on the right-hand side of Eq. (3), we first calculate the commutator $[\Pi_m(t), \Pi_n(t)]$ $(m, n = x, y, z)$ between two components of $\mathbf{\Pi}(t)$:

$$\begin{aligned} [\Pi_m(t), \Pi_n(t)] =& [p_m(t) - A_m(t), p_n(t) - A_n(t)] \\ =& - i\hbar\frac{\partial A_m(t)}{\partial x_n} + i\hbar\frac{\partial A_n(t)}{\partial x_m} + [A_m(t)A_n(t) - A_n(t)A_m(t)] \\ =& i\hbar\epsilon_{lmn}B_l(t), \end{aligned} \tag{4}$$

where ϵ_{lmn} is the Levi-Civita tensor and the generalized non-Abelian magnetic field is given by

$$\mathbf{B}(t) = \nabla \times \mathbf{A}(t) - \frac{i}{\hbar}\mathbf{A}(t) \times \mathbf{A}(t). \tag{5}$$

By using Eq. (4), we obtain the commutator between $\mathbf{\Pi}(t)$ and the first term in the Hamiltonian (1) as

$$\begin{aligned} [\mathbf{\Pi}(t), H_1] =& \frac{i\hbar}{2M}[\mathbf{\Pi}(t) \times \mathbf{B}(t) - \mathbf{B}(t) \times \mathbf{\Pi}(t)] \\ =& \frac{1}{2}\left[\frac{\mathrm{d}\mathbf{r}}{\mathrm{d}t} \times \mathbf{B}(t) - \mathbf{B}(t) \times \frac{\mathrm{d}\mathbf{r}}{\mathrm{d}t}\right]. \end{aligned} \tag{6}$$

This term can be interpreted as a generalized Lorentz force exerted by the magnetic field. For the emergent gauge potential generated by the helical spin texture, $\check{\mathbf{A}}(\mathbf{r}) = \mathbf{A}^x\check{\sigma}_x + \mathbf{A}^y\check{\sigma}_y + \mathbf{A}^z\check{\sigma}_z$ with $\mathbf{A}^x = -\boldsymbol{\kappa}\cos(\boldsymbol{\kappa}\cdot\mathbf{r})/2$, $\mathbf{A}^y = -\boldsymbol{\kappa}\sin(\boldsymbol{\kappa}\cdot\mathbf{r})/2$, and $\mathbf{A}^z = \boldsymbol{\kappa}/2$ (see Eqs. (4)–(6) in the main text). Substituting these in Eq. (5), we find that $\mathbf{B} = 0$.

Next, we evaluate the commutator between $\mathbf{\Pi}(t)$ and the second term in the Hamiltonian (1):

$$\begin{aligned} [\mathbf{\Pi}(t), H_2] =& -\frac{J}{M}e^{iHt/\hbar}[\hat{\mathbf{p}}\check{1} - \check{\mathbf{A}}(\hat{\mathbf{r}}), \hat{1}\check{\sigma}_z]e^{-iHt/\hbar} \\ =& \frac{J}{M}e^{iHt/\hbar}[\check{\mathbf{A}}(\hat{\mathbf{r}}), \hat{1}\check{\sigma}_z]e^{-iHt/\hbar}, \end{aligned} \tag{7}$$

where $\check{\mathbf{A}}(\hat{\mathbf{r}}) = \mathbf{A}^x(\hat{\mathbf{r}})\check{\sigma}_x + \mathbf{A}^y(\hat{\mathbf{r}})\check{\sigma}_y + \mathbf{A}^z(\hat{\mathbf{r}})\check{\sigma}_z$.

Due to the non-Abelianness, i.e., $[\check{\sigma}_m, \check{\sigma}_n] = i\epsilon_{lmn}\check{\sigma}_l$, in the gauge potential $\check{\mathbf{A}}$, the right-hand side of Eq. (7) is non-vanishing, and the equation of motion (3) leads to

$$\begin{aligned}\frac{\mathrm{d}^2\mathbf{r}}{\mathrm{d}t^2} =& \frac{J}{M^2} e^{iHt/\hbar}[\mathbf{A}_y(\hat{\mathbf{r}})\check{\sigma}_x - \mathbf{A}_x(\hat{\mathbf{r}})\check{\sigma}_y] e^{-iHt/\hbar} \\ =& \frac{J\boldsymbol{\kappa}}{2M^2} e^{iHt/\hbar}[\cos(\boldsymbol{\kappa}\cdot\hat{\mathbf{r}})\check{\sigma}_y - \sin(\boldsymbol{\kappa}\cdot\hat{\mathbf{r}})\check{\sigma}_x] e^{-iHt/\hbar}.\end{aligned} \tag{8}$$

Since the right-hand side of Eq. (8) depends only on $\hat{\mathbf{r}}$ and $\check{\boldsymbol{\sigma}}$ (not on $\mathrm{d}\hat{\mathbf{r}}/\mathrm{d}t$), it can be interpreted as a force exerted on the particle by a generalized non-Abelian electric field. It is this generalized electric field that generates a pure spin current of fermionic atoms in the mixture as discussed in the main text. It is also evident from Eq. (8) that the orientation of the generalized electric field is parallel to that of the wavevector $\boldsymbol{\kappa}$ of the helical spin texture and thus it is parallel to that of the pure spin current $\mathbf{j}_{\mathrm{s}}^z$.

SUPPLEMENTARY NOTE 2: NON-ABELIAN CONTRIBUTION OF THE EMERGENT GAUGE POTENTIAL TO THE SPIN CURRENT DENSITY

In this section we derive the non-Abelian contribution of the emergent gauge potential to the spin current density. As shown in Eq. (9) in the main text, the z-axis spin-polarization component $\mathbf{j}_{\mathrm{s}}^{z(\mathrm{NA})}$ of the spin current density in the laboratory frame can be expressed as a linear superposition of the spin current densities $\tilde{\mathbf{j}}_{\mathrm{s}}^x$ and $\tilde{\mathbf{j}}_{\mathrm{s}}^y$ in the adiabatic frame.

To evaluate $\tilde{\mathbf{j}}_{\mathrm{s}}^x$ and $\tilde{\mathbf{j}}_{\mathrm{s}}^y$, we use the non-equilibrium Green's function method. The spin current density $\tilde{\mathbf{j}}_{\mathrm{s}}^\alpha$ $(\alpha = x, y)$ can be expressed in terms of the lesser Green's function by Eq. (19) in the Methods section. On the other hand, the lesser Green's function is related to the Keldysh Green's function by taking the two arguments on the forward and backward parts of the Keldysh contour [1]. Making a perturbative expansion of the Dyson equation with respect to the emergent gauge potential, we obtain the Fourier transform $\tilde{G}^<_{\mathbf{k},\mathbf{k}',\omega}$ of the lesser Green's function up to the linear order in $\mathbf{A}^\alpha$ as given by Eq. (10) in the main text. Substituting it in Eq. (19) in the Methods section, we obtain

$$\tilde{\mathbf{j}}_{\mathrm{s}}^\alpha(\mathbf{r}) = -\frac{i\hbar^2}{4\pi M_{\mathrm{f}}^2 V}\sum_{\mathbf{q},\mathbf{p},\beta}\int d\omega\; e^{-i\mathbf{p}\cdot\mathbf{r}}\mathbf{q}\Big[\mathbf{q}\cdot\mathbf{A}_{\mathbf{p}}^\beta\Big]\mathrm{Tr}\Big\{\sigma_\alpha\tilde{g}_{\mathbf{q}-\frac{\mathbf{p}}{2},\omega}\sigma_\beta\tilde{g}_{\mathbf{q}+\frac{\mathbf{p}}{2},\omega}\Big\}^<, \tag{9}$$

where $\mathbf{q} \equiv \frac{\mathbf{k}+\mathbf{k}'}{2}$ and $\mathbf{p} \equiv \mathbf{k}' - \mathbf{k}$. Here the non-interacting Green's function $\tilde{g}$ is a 2×2 diagonal matrix. The trace over the spin indices on the right-hand side of Eq. (9) can be evaluated by using

$$\mathrm{Tr}\Big\{\sigma_\alpha D_1\sigma_\beta D_2\Big\} = (d_{1-}d_{2+} + d_{1+}d_{2-})\delta_{\alpha\beta} + (d_{1+} - d_{1-})(d_{2+} - d_{2-})\delta_{\alpha z}\delta_{\beta z} + i(d_{1-}d_{2+} - d_{1+}d_{2-})\epsilon_{\alpha\beta z}, \tag{10}$$

where $D_1 = \mathrm{diag}(d_{1+}, d_{1-})$ and $D_2 = \mathrm{diag}(d_{2+}, d_{2-})$ are diagonal matrices. For the helical spin texture, $\mathbf{A}^\pm(\mathbf{r}) \equiv \mathbf{A}^x \pm i\mathbf{A}^y = -\boldsymbol{\kappa}e^{\pm i\boldsymbol{\kappa}\cdot\mathbf{r}}/2$, or equivalently, $\mathbf{A}_{\mathbf{p}}^\pm = -\boldsymbol{\kappa}\delta_{\mathbf{p},\mp\boldsymbol{\kappa}}/2$. Substituting Eq. (9) in this section in Eq. (9) in the main text, the non-Abelian contribution to the spin current density is obtained as

$$\mathbf{j}_{\mathrm{s}}^{z(\mathrm{NA})} = \frac{i\hbar^2}{4\pi M_{\mathrm{f}}^2 V}\sum_{\mathbf{q}}\int d\omega\;\mathbf{q}(\mathbf{q}\cdot\boldsymbol{\kappa})\Big[\tilde{g}_{-,\mathbf{q}+\frac{\boldsymbol{\kappa}}{2},\omega}\tilde{g}_{+,\mathbf{q}-\frac{\boldsymbol{\kappa}}{2},\omega}\Big]^<. \tag{11}$$

Here $\tilde{g}_\pm$ denotes the non-interacting Green's functions of particles with spin up (+) and spin down (-) in the adiabatic frame. It should be noted that the factor of $e^{-i\mathbf{p}\cdot\mathbf{r}}$ on the right-hand side of Eq. (9) is cancelled by the coefficients of $\cos(\boldsymbol{\kappa}\cdot\mathbf{r})$ and $\sin(\boldsymbol{\kappa}\cdot\mathbf{r})$ in Eq. (9) in the main text due to the Kronecker delta $\delta_{\mathbf{p},\mp\boldsymbol{\kappa}}$ from $\mathbf{A}_{\mathbf{p}}^\pm$, resulting in the $\mathbf{r}$-independence of $\mathbf{j}_{\mathrm{s}}^{z(\mathrm{NA})}$ as shown in Eq. (11). The lesser component of the product of Green's functions on the right-hand side of Eq. (11) can be expressed in terms of the retarded and advanced components of the constituent Green's functions by using the Langreth rule $[\tilde{g}\tilde{g}]^< = \tilde{g}^{\mathrm{r}}\tilde{g}^< + \tilde{g}^<\tilde{g}^{\mathrm{a}}$ [1]. The retarded, advanced, and lesser components

of the non-interacting Green's function are respectively given by

$$\tilde{g}^{\mathrm{r}}_{\pm,\mathbf{q},\omega} = \frac{1}{\omega - \frac{\epsilon_{\mathbf{q}}-\mu_\pm}{\hbar} + i\eta}, \tag{12}$$

$$\tilde{g}^{\mathrm{a}}_{\pm,\mathbf{q},\omega} = \frac{1}{\omega - \frac{\epsilon_{\mathbf{q}}-\mu_\pm}{\hbar} - i\eta}, \tag{13}$$

$$\tilde{g}^{<}_{\pm,\mathbf{q},\omega} = 2\pi i f(\omega)\delta\left(\omega - \frac{\epsilon_{\mathbf{q}}-\mu_\pm}{\hbar}\right), \tag{14}$$

where $\epsilon_{\mathbf{q}} \equiv \hbar^2\mathbf{q}^2/(2M_{\mathrm{f}})$, $\mu_\pm \equiv \mu \mp J$, $f(\omega) = \left[e^{\hbar\omega/(k_{\mathrm{B}}T)}+1\right]^{-1}$ is the Fermi-Dirac distribution function, and η is an infinitesimal positive number. The integral over ω in Eq. (11) then yields $2\pi i\hbar\left[f(\epsilon_{\mathbf{q}-\frac{\boldsymbol{\kappa}}{2}}-\mu_+) - f(\epsilon_{\mathbf{q}+\frac{\boldsymbol{\kappa}}{2}}-\mu_-)\right]/(\epsilon_{\mathbf{q}-\frac{\boldsymbol{\kappa}}{2}} - \epsilon_{\mathbf{q}+\frac{\boldsymbol{\kappa}}{2}} + \mu_- - \mu_+)$. For a smooth spatial variation of the spin texture ($\kappa \ll k_{\mathrm{F}}$), we can approximate $\mathbf{q} \pm \frac{\boldsymbol{\kappa}}{2} \simeq \mathbf{q}$, and thus $\mathbf{j}_{\mathrm{s}}^{z(\mathrm{NA})}$ is found to be

$$\mathbf{j}_{\mathrm{s}}^{z(\mathrm{NA})} = -\frac{\hbar^3\boldsymbol{\kappa}}{12M_{\mathrm{f}}^2VJ}\sum_{\mathbf{q}}|\mathbf{q}|^2\left[\tilde{f}_+(\mathbf{q}) - \tilde{f}_-(\mathbf{q})\right], \tag{15}$$

where $\tilde{f}_\pm(\mathbf{q}) \equiv f(\epsilon_{\mathbf{q}} - \mu_\pm)$ are the distribution functions of particles with spin up (+) and spin down (-) in the adiabatic frame. The difference between μ_+ and μ_- arises from the effective magnetic Zeeman energy (the last term in Eq. (3) in the main text). Therefore, the non-Abelian contribution to the spin current density is proportional to the difference in the total kinetic energy density between particles with spin up and down in the adiabatic frame as shown on the right-hand side of Eq. (11) in the main text.

SUPPLEMENTARY NOTE 3: SPIN CURRENT DENSITY IN THE WEAK-COUPLING LIMIT

In the weak-coupling limit ($J \ll \epsilon_{\mathrm{F}}$), where the perturbative expansion with respect to $\mathbf{A}^\alpha$ does not converge, we can alternatively make an expansion of the spin current density in powers of J/ϵ_{F} in the laboratory frame. Up to the second order, the spin current density is given by

$$\begin{aligned}\mathbf{j}_{\mathrm{s}}^{\alpha}(\mathbf{r},t) = &-\frac{\hbar(\nabla_{\mathbf{r}}-\nabla_{\mathbf{r}'})}{4M_{\mathrm{f}}}\Bigg\{\mathrm{Tr}\left\{\sigma_\alpha\right\}g^<(\mathbf{r},\mathbf{r}',t,t') + \frac{J}{\hbar}\int \mathrm{d}t_1\int \mathrm{d}^3\mathbf{r}_1\ \mathrm{Tr}\Big\{\sigma_\alpha\left[\mathbf{F}(\mathbf{r}_1)\cdot\boldsymbol{\sigma}\right]\Big\}\Big[g(\mathbf{r},\mathbf{r}_1,t,t_1)g(\mathbf{r}_1,\mathbf{r}',t_1,t')\Big]^<\\ &+\left(\frac{J}{\hbar}\right)^2\int \mathrm{d}t_1\int \mathrm{d}t_2\int \mathrm{d}^3\mathbf{r}_1\int \mathrm{d}^3\mathbf{r}_2\ \mathrm{Tr}\Big\{\sigma_\alpha\left[\mathbf{F}(\mathbf{r}_1)\cdot\boldsymbol{\sigma}\right]\left[\mathbf{F}(\mathbf{r}_2)\cdot\boldsymbol{\sigma}\right]\Big\}\\ &\times\Big[g(\mathbf{r},\mathbf{r}_1,t,t_1)g(\mathbf{r}_1,\mathbf{r}_2,t_1,t_2)g(\mathbf{r}_2,\mathbf{r}',t_2,t')\Big]^<\Bigg\}\Bigg|_{\mathbf{r}'\to\mathbf{r},t'\to t},\end{aligned} \tag{16}$$

where the second-order term is illustrated in terms of the Feynman diagram in Fig. 1 of this Supplementary Note. Since $\mathrm{Tr}\{\sigma_\alpha\} = 0$, and $\mathrm{Tr}\Big\{\sigma_\alpha[\mathbf{F}(\mathbf{r}_1)\cdot\boldsymbol{\sigma}]\Big\} = 2F_\alpha(\mathbf{r}_1)$ and $F_z = 0$ for the helical spin texture, the zeroth- and first-order contributions in Eq. (16) to the z-axis spin-polarization component $\mathbf{j}_{\mathrm{s}}^z$ vanish. Using the relation

$$\mathrm{Tr}\Big\{\sigma_\alpha[\mathbf{F}(\mathbf{r}_1)\cdot\boldsymbol{\sigma}][\mathbf{F}(\mathbf{r}_2)\cdot\boldsymbol{\sigma}]\Big\} = 2i[\mathbf{F}(\mathbf{r}_1)\times\mathbf{F}(\mathbf{r}_2)]_\alpha \tag{17}$$

and making a Fourier transformation of the second-order term on the right-hand side of Eq. (16), we obtain the spin current density $\mathbf{j}_{\mathrm{s}}^z$ as given by Eq. (11) in the main text.

For the helical spin texture, we have $F_\pm(\mathbf{r}) \equiv F_x \pm iF_y = e^{i\boldsymbol{\kappa}\cdot\mathbf{r}}$ or $F^\pm_{\mathbf{q}} = \delta_{\mathbf{q},\mp\boldsymbol{\kappa}}$, where $\mathbf{F}_{\mathbf{q}} \equiv (1/V)\int \mathrm{d}^3\mathbf{r}\, e^{i\mathbf{q}\cdot\mathbf{r}}\mathbf{F}(\mathbf{r})$ with V being the volume of the system. Applying the Langreth rule to the lesser component of the product of Green's functions $[ggg]^<$ and using the relation $g^<_{\mathbf{k},\omega} = f(\omega)\Big[g^{\mathrm{a}}_{\mathbf{k},\omega} - g^{\mathrm{r}}_{\mathbf{k},\omega}\Big]$ between the lesser, retarded, and advanced components of the non-interacting Green's function, $\mathbf{j}_{\mathrm{s}}^z$ can be rewritten as

$$\mathbf{j}_{\mathrm{s}}^z = -\frac{iJ^2}{4\pi\hbar M_{\mathrm{f}}V}\sum_{\mathbf{k}}\mathbf{k}\int_{-\infty}^{\infty}\mathrm{d}\omega f(\omega)\Big[\left(g^{\mathrm{a}}_{\mathbf{k},\omega}\right)^2\left(g^{\mathrm{a}}_{\mathbf{k}+\boldsymbol{\kappa},\omega} - g^{\mathrm{a}}_{\mathbf{k}-\boldsymbol{\kappa},\omega}\right) - \left(g^{\mathrm{r}}_{\mathbf{k},\omega}\right)^2\left(g^{\mathrm{r}}_{\mathbf{k}+\boldsymbol{\kappa},\omega} - g^{\mathrm{r}}_{\mathbf{k}-\boldsymbol{\kappa},\omega}\right)\Big]. \tag{18}$$

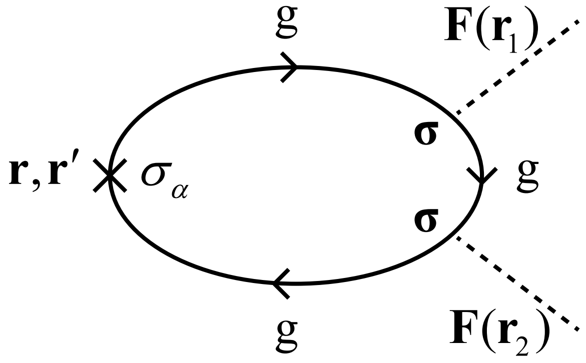

FIG. 1: **Feynman diagram for the second-order contribution to the spin current density of a Bose-Fermi mixture in the weak-coupling limit**. Fermions, which are represented by Green's function g (solid curves), interact twice at points $\mathbf{r}_1$ and $\mathbf{r}_2$ with the spin texture of the Bose-Einstein condensate whose magnetization $\mathbf{F}(\mathbf{r})$ is represented by the dashed lines. The Pauli matrix σ_α ($\alpha = x, y, z$) is due to the α-polarization component of the spin current, while $\boldsymbol{\sigma}$ arises from the spin-dependent interaction between bosons and fermions. As shown in Eq. (16), the spin current density is obtained by taking the difference in the spatial gradient between $\mathbf{r}$ and $\mathbf{r}'$, followed by taking the limit of $\mathbf{r}' \to \mathbf{r}$.

The pitch of the helical spin texture can be adjusted by varying the duration of the applied magnetic-field gradient. We consider the typical case of a smooth spatial variation of the spin texture ($\kappa \ll k_{\mathrm{F}}$), for which we can make a Taylor expansion of the Green's functions around $\kappa = 0$ and ignore quadratic and higher-order terms:

$$g^{\mathrm{r,a}}_{\mathbf{k}\pm\boldsymbol{\kappa},\omega} \simeq g^{\mathrm{r,a}}_{\mathbf{k},\omega} \pm \left(g^{\mathrm{r,a}}_{\mathbf{k},\omega}\right)^2 \frac{\hbar\mathbf{k}\cdot\boldsymbol{\kappa}}{M_{\mathrm{f}}}. \tag{19}$$

Here we used the expressions $g^{\mathrm{r}}_{\mathbf{k},\omega} = \left(\omega - \frac{\epsilon_{\mathbf{k}}-\mu}{\hbar} + i\eta\right)^{-1}$ and $g^{\mathrm{a}}_{\mathbf{k},\omega} = (g^{\mathrm{r}}_{\mathbf{k},\omega})^*$, from which $\partial g^{\mathrm{r,a}}_{\mathbf{k},\omega}/\partial\epsilon_{\mathbf{k}} = \left(g^{\mathrm{r,a}}_{\mathbf{k},\omega}\right)^2/\hbar$. The spin current density then reduces to

$$\begin{aligned}\mathbf{j}^z_{\mathrm{s}} =& -\frac{iJ^2}{2\pi M_{\mathrm{f}}^2 V}\sum_{\mathbf{k}} \mathbf{k}(\mathbf{k}\cdot\boldsymbol{\kappa}) \int_{-\infty}^{\infty} \mathrm{d}\omega\, f(\omega)\left[\left(g^{\mathrm{a}}_{\mathbf{k},\omega}\right)^4 - \left(g^{\mathrm{r}}_{\mathbf{k},\omega}\right)^4\right] \\ =& -\frac{iJ^2\boldsymbol{\kappa}}{6\pi M_{\mathrm{f}}^2 V}\sum_{\mathbf{k}} |\mathbf{k}|^2 \int_{-\infty}^{\infty} \mathrm{d}\omega\; f(\omega)\left[\left(g^{\mathrm{a}}_{\mathbf{k},\omega}\right)^4 - \left(g^{\mathrm{r}}_{\mathbf{k},\omega}\right)^4\right].\end{aligned} \tag{20}$$

Here in the second equation we used the fact that $g^{\mathrm{r,a}}_{\mathbf{k},\omega}$ depends only on $|\mathbf{k}|$, and $\sum_{\mathbf{k}} k_x^2 = \sum_{\mathbf{k}} k_y^2 = \sum_{\mathbf{k}} k_z^2 = (1/3)\sum_{\mathbf{k}} |\mathbf{k}|^2$. Replacing the sum over $\mathbf{k}$ in Eq. (20) by the corresponding integral over the energy $\epsilon \equiv \epsilon_{\mathbf{k}} = \hbar^2\mathbf{k}^2/(2M)$, we obtain

$$\mathbf{j}^z_{\mathrm{s}} = \frac{iJ^2 M_{\mathrm{f}}^{1/2}\boldsymbol{\kappa}}{3\sqrt{2}\pi^3\hbar} \int_{-\infty}^{\infty} \mathrm{d}\omega\, \frac{1}{e^{\beta\hbar\omega}+1} \int_0^{\infty} \mathrm{d}\epsilon\, \epsilon^{3/2}\left[\frac{1}{(\hbar\omega-\epsilon+\mu+i\eta)^4} - \frac{1}{(\hbar\omega-\epsilon+\mu-i\eta)^4}\right], \tag{21}$$

where $\beta \equiv 1/(k_{\mathrm{B}}T)$ and we used the explicit expressions for the Fermi-Dirac distribution function $f(\omega)$ and Green's functions $g^{\mathrm{r,a}}_{\mathbf{k},\omega}$.

To obtain the spin current density at zero temperature, we divide the integration domain of ω in Eq. (21) into two parts $\int_{-\infty}^0 \mathrm{d}\omega$ and $\int_0^{\infty} \mathrm{d}\omega$, and use the identity

$$\int_{-\infty}^{0} \mathrm{d}\omega\; g(\omega)\frac{1}{e^{\beta\hbar\omega}+1} = \int_0^{\infty} \mathrm{d}\omega\; g(-\omega)\left(1 - \frac{1}{e^{\beta\hbar\omega}+1}\right), \tag{22}$$

which holds for an arbitrary function $g(\omega)$. The spin current density at $T=0$ is then given by

$$\begin{aligned}
\mathbf{j}_{\rm s}^z(T=0) =& \frac{iJ^2 M_{\rm f}^{1/2}\boldsymbol{\kappa}}{3\sqrt{2}\pi^3\hbar}\int_0^\infty \mathrm{d}\omega \int_0^\infty \mathrm{d}\epsilon\, \epsilon^{3/2}\left[\frac{1}{(-\hbar\omega-\epsilon+\mu+i\eta)^4}-\frac{1}{(-\hbar\omega-\epsilon+\mu-i\eta)^4}\right] \\
=& -\frac{J^2 M_{\rm f}^{1/2}\boldsymbol{\kappa}}{24\pi^2\hbar^2\mu^{1/2}}\frac{\sqrt{1+\sqrt{1+\tilde{\eta}^2}}}{\sqrt{1+\tilde{\eta}^2}},
\end{aligned} \tag{23}$$

where $\tilde{\eta}\equiv\eta/\mu$. By setting $\mu=\epsilon_{\rm F}$ at $T=0$ and taking the limit $\eta\to 0$, we obtain

$$\begin{aligned}
\mathbf{j}_{\rm s}^z(T=0) =& -\frac{J^2 M_{\rm f}^{1/2}\boldsymbol{\kappa}}{12\sqrt{2}\pi^2\hbar^2\epsilon_{\rm F}^{1/2}} \\
=& -\frac{\hbar n_{\rm f} J^2\boldsymbol{\kappa}}{16 M_{\rm f}\epsilon_{\rm F}^2}.
\end{aligned} \tag{24}$$

Here, we used $n_{\rm f}=2\sum_{|\mathbf{k}|<k_{\rm F}}=2\sqrt{2}M_{\rm f}^{3/2}\epsilon_{\rm F}^{3/2}/(3\pi^2\hbar^3)$ in deriving the second equality.

It follows from Eqs. (21) and (22) that the temperature dependence of the spin current density is given by

$$\begin{aligned}
\mathbf{j}_{\rm s}^z(T)-\mathbf{j}_{\rm s}^z(T=0) =& \frac{iJ^2 M_{\rm f}^{1/2}\boldsymbol{\kappa}}{3\sqrt{2}\pi^3\hbar}\int_0^\infty \mathrm{d}\omega\,\frac{1}{e^{\beta\hbar\omega}+1}\int_0^\infty \mathrm{d}\epsilon\,\epsilon^{3/2}\left[\frac{1}{(\hbar\omega-\epsilon+\mu+i\eta)^4}-\frac{1}{(\hbar\omega-\epsilon+\mu-i\eta)^4}\right. \\
&\left.-\frac{1}{(\hbar\omega+\epsilon-\mu-i\eta)^4}+\frac{1}{(\hbar\omega+\epsilon-\mu+i\eta)^4}\right].
\end{aligned} \tag{25}$$

We first consider the low-temperature regime $k_{\rm B}T\ll\epsilon_{\rm F}$. Since the dominant contribution to the integral $\int \mathrm{d}\omega$ in Eq. (25) is given by $\omega\sim\epsilon_{\rm F}$, we can take a series expansion of the Fermi-Dirac distribution function for $\beta\hbar\omega\ll 1$ as

$$\frac{1}{e^{\beta\hbar\omega}+1}=\sum_{n=1}^\infty(-1)^{n-1}e^{-n\beta\hbar\omega}. \tag{26}$$

The integral over ω on the right-hand side of Eq. (25) is then rewritten as

$$\int_0^\infty \mathrm{d}\omega\,\frac{1}{(e^{\beta\hbar\omega}+1)\,(\hbar\omega-\epsilon+\mu+i\eta)^4}=\sum_{n=1}^\infty(-1)^{n-1}\int_0^\infty \mathrm{d}\omega\,\frac{e^{-n\beta\hbar\omega}}{(\hbar\omega-\epsilon+\mu+i\eta)^4}. \tag{27}$$

Using the partial integration, the integral on the right-hand side of Eq. (27) can be calculated in a straightforward manner, yielding

$$\begin{aligned}
\int_0^\infty \mathrm{d}x\,\frac{e^{-ax}}{(x+b)^4} =& \frac{1}{3b^3}-\frac{a}{6b^2}+\frac{a^2}{6b}-\frac{a^3}{6}\int_0^\infty \mathrm{d}x\,\frac{e^{-ax}}{x+b} \\
=& \frac{1}{3b^3}-\frac{a}{6b^2}+\frac{a^2}{6b}-\frac{a^3}{6}e^{ab}\Gamma(0,ab),
\end{aligned} \tag{28}$$

where $\Gamma(\lambda,z)\equiv\int_z^\infty \mathrm{d}t\; t^{\lambda-1}e^{-t}$ is the incomplete Gamma function. The asymptotic expansion of this function in the limit of $a\to\infty$ with b held fixed is given by

$$\Gamma(0,ab)=e^{-ab}\left[\frac{1}{ab}-\frac{1}{(ab)^2}+\frac{2}{(ab)^3}-\frac{6}{(ab)^4}+\frac{24}{(ab)^5}+\mathcal{O}\left(\frac{1}{(ab)^6}\right)\right]. \tag{29}$$

Substituting this in Eq. (28), we have

$$\int_0^\infty \mathrm{d}x\,\frac{e^{-ax}}{(x+b)^4}=\frac{1}{ab^4}-\frac{4}{a^2b^5}+\mathcal{O}\left(\frac{1}{a^3b^6}\right). \tag{30}$$

The left-hand side of Eq. (27) is then given up to the order of $T^2 \propto \beta^{-2}$ as

$$\int_0^\infty \mathrm{d}\omega \, \frac{1}{\left(e^{\beta\hbar\omega}+1\right)\left(\hbar\omega-\epsilon+\mu+i\eta\right)^4} = \frac{1}{\hbar\beta}\sum_{n=1}^{\infty}\frac{(-1)^{n-1}}{n}\frac{1}{\left(-\epsilon+\mu+i\eta\right)^4} - \frac{4}{\hbar\beta^2}\sum_{n=1}^{\infty}\frac{(-1)^{n-1}}{n^2}\frac{1}{\left(-\epsilon+\mu+i\eta\right)^5}$$
$$= \frac{\ln 2}{\hbar\beta}\frac{1}{\left(-\epsilon+\mu+i\eta\right)^4} - \frac{\pi^2}{3\hbar\beta^2}\frac{1}{\left(-\epsilon+\mu+i\eta\right)^5}, \tag{31}$$

where we used $\sum_{n=1}^{\infty}(-1)^{n-1}/n = \ln 2$ and $\sum_{n=1}^{\infty}(-1)^{n-1}/n^2 = \pi^2/12$. It is clear that the first term on the right-hand side of Eq. (31) will be cancelled as we take the sum inside the square bracket in Eq. (25). On the other hand, the second term on the right-hand side of Eq. (31) gives a nonvanishing contribution to the spin current density at low temperature as

$$\mathbf{j}_{\mathrm{s}}^z(T) - \mathbf{j}_{\mathrm{s}}^z(T=0) = -\frac{i\sqrt{2}J^2 M_{\mathrm{f}}^{1/2}\boldsymbol{\kappa}}{9\pi\hbar^2\beta^2}\int_0^\infty \mathrm{d}\epsilon \, \epsilon^{3/2}\left[\frac{1}{\left(-\epsilon+\mu+i\eta\right)^5} - \frac{1}{\left(-\epsilon+\mu-i\eta\right)^5}\right]$$
$$= \frac{J^2 M_{\mathrm{f}}^{1/2}\boldsymbol{\kappa}\left[(\tilde{\eta}^3-3\tilde{\eta}^2)\sqrt{\sqrt{1+\tilde{\eta}^2}-1} + (3\tilde{\eta}^2-1)\sqrt{\sqrt{1+\tilde{\eta}^2}+1}\right]}{192\hbar^2\mu^{5/2}\beta^2(\tilde{\eta}^2+1)^3}, \tag{32}$$

where $\tilde{\eta} \equiv \eta/\mu$. Taking the limit $\eta \to 0$, we obtain the temperature dependence of the spin current density in the low-temperature regime as

$$\mathbf{j}_{\mathrm{s}}^z(T) - \mathbf{j}_{\mathrm{s}}^z(T=0) = -\frac{J^2 M^{1/2}(k_{\mathrm{B}}T)^2\boldsymbol{\kappa}}{96\sqrt{2}\hbar^2\mu^{5/2}}$$
$$\simeq -\frac{J^2 M^{1/2}(k_{\mathrm{B}}T)^2\boldsymbol{\kappa}}{96\sqrt{2}\hbar^2\epsilon_{\mathrm{F}}^{5/2}}$$
$$= -\frac{\pi^2\hbar n_{\mathrm{f}}J^2(k_{\mathrm{B}}T)^2\boldsymbol{\kappa}}{128M_{\mathrm{f}}\epsilon_{\mathrm{F}}^4}, \tag{33}$$

where in the second equation we have replaced the chemical potential μ at low temperatures by the Fermi energy ϵ_{F} since their difference gives rise to a correction that is beyond the order of magnitude under consideration. Combining Eqs. (24) and (33), we find that the spin current density at low temperature $k_{\mathrm{B}}T \ll \epsilon_{\mathrm{F}}$ is given by

$$\mathbf{j}_{\mathrm{s}}^z(T) = -\frac{\hbar n_{\mathrm{f}}J^2\boldsymbol{\kappa}}{16M_{\mathrm{f}}\epsilon_{\mathrm{F}}^2}\left[1+\frac{\pi^2}{8}\left(\frac{k_{\mathrm{B}}T}{\epsilon_{\mathrm{F}}}\right)^2\right]. \tag{34}$$

We have thus derived Eq. (15) in the main text. As the temperature dependence of the coupling constant J via the density of condensate particles $n_{\mathrm{b}}(T)$ is taken into account, we arrive at Eq. (16) in the main text for the temperature dependence of the spin current density j_{s}^z.

For higher temperatures, we numerically evaluate the spin current density given by Eq. (21). The value of the chemical potential μ as a function of temperature for a given particle-number density is obtained by numerically solving the equation

$$n_{\mathrm{f}} = 2\sum_{\mathbf{k}} f_{\mathbf{k}}$$
$$= \frac{1}{\pi^2}\int_0^\infty \mathrm{d}k \, k^2 \frac{1}{e^{\beta(\epsilon_{\mathbf{k}}-\mu)}+1}. \tag{35}$$

The result is shown in Fig. 3 in the main text. The initial growth of the spin current density with increasing temperature in the low-temperature regime agrees with the analytic result [i.e., Eq. (16) in the main text]. It is attributed to the expansion of the Fermi-Dirac distribution towards the states with high velocities due to thermal excitations. In contrast, at high temperatures where the Fermi gas becomes non-degenerate, thermal random motion of fermions and a decrease in the number of condensate particles suppress the directional flow of the spin current. The maximum value of j_{s}^z is attained at the temperature given by $k_{\mathrm{B}}T_{\max} \simeq 0.3\,\epsilon_{\mathrm{F}}$.

SUPPLEMENTARY NOTE 4: s-WAVE SCATTERING LENGTHS IN THE HYPERFINE SPIN BASIS

As shown in Eq. (14) in the main text, the magnitude of the coupling constant J between fermions and the spin texture is proportional to the difference $a_{S+\frac{1}{2}} - a_{S-\frac{1}{2}}$ between the s-wave scattering lengths for two total-hyperfine-spin-F channels: $F = S \pm 1/2$. For a mixture of a spin-1 ^{87}Rb Bose-Einstein condensate and a ^{6}Li Fermi gas with spin-1/2, we need to evaluate the magnitude of $|a_{3/2} - a_{1/2}|$. On the other hand, the electronic spin-singlet and spin-triplet atomic potentials for the ^{87}Rb-^{6}Li mixture as well as the corresponding s-wave scattering lengths $a_{\rm s}$ and $a_{\rm t}$ have been calculated numerically in Refs. [2] and [3]. In this section, by expanding the hyperfine-spin states in the electronic spin bases, we obtain the atomic potentials V_F associated with the total-hyperfine-spin-F channels ($F = 3/2$ and $1/2$) for the ^{87}Rb-^{6}Li mixture, from which we can evaluate the corresponding scattering lengths. Without a strong external field the atomic interaction is almost isotropic, and thus the inter-atomic potential is essentially the same for all Zeeman states $|F, m_F\rangle$ in a given total-hyperfine-spin-F manifold. Therefore, we only need to calculate the atomic potential for an arbitrary hyperfine state within that spin manifold.

First, we expand the hyperfine-spin state $|F = \frac{3}{2}, m_F = \frac{3}{2}\rangle$ in the electronic-spin basis. In terms of the hyperfine spins $F_1 = 1$ for the boson and $F_2 = 1/2$ for the fermion, that state can be written as

$$|F_1 = 1, m_{F_1} = 1\rangle \otimes \left|F_2 = \frac{1}{2}, m_{F_2} = \frac{1}{2}\right\rangle. \tag{36}$$

Since both rubidium and lithium are alkali atoms whose valence electron has zero orbital angular momentum ($l = 0$), their hyperfine spins are the sums of the electronic spin s and the nucleus spin I. Here we have $I_1 = \frac{3}{2}$, $s_1 = \frac{1}{2}$ and $I_2 = 1$, $s_2 = \frac{1}{2}$ for ^{87}Rb and ^{6}Li, respectively. Using the table of Clebsch-Gordan coefficients [4], the hyperfine states in Eq. (36) can be expressed as

$$|F_1 = 1, m_{F_1} = 1\rangle = \sqrt{\frac{3}{4}}\left|m_{I_1} = \frac{3}{2}, m_{s_1} = -\frac{1}{2}\right\rangle - \frac{1}{2}\left|m_{I_1} = \frac{1}{2}, m_{s_1} = \frac{1}{2}\right\rangle, \tag{37}$$

$$\left|F_2 = \frac{1}{2}, m_{F_2} = \frac{1}{2}\right\rangle = \sqrt{\frac{2}{3}}\left|m_{I_2} = 1, m_{s_1} = -\frac{1}{2}\right\rangle - \sqrt{\frac{1}{3}}\left|m_{I_2} = 0, m_{s_2} = \frac{1}{2}\right\rangle. \tag{38}$$

Substituting Eqs. (37) and (38) in Eq. (36), we can express the hyperfine state $|F = \frac{3}{2}, m_F = \frac{3}{2}\rangle$ in the electronic-spin basis as

$$\begin{aligned}\left|F = \frac{3}{2}, m_F = \frac{3}{2}\right\rangle =& \sqrt{\frac{1}{2}}\left|m_{s_1} = -\frac{1}{2}, m_{s_2} = -\frac{1}{2}\right\rangle - \sqrt{\frac{1}{6}}\left|m_{s_1} = \frac{1}{2}, m_{s_2} = -\frac{1}{2}\right\rangle - \frac{1}{2}\left|m_{s_1} = -\frac{1}{2}, m_{s_2} = \frac{1}{2}\right\rangle \\ &+ \sqrt{\frac{1}{12}}\left|m_{s_1} = \frac{1}{2}, m_{s_2} = \frac{1}{2}\right\rangle \\ =& \sqrt{\frac{1}{2}}|s = 1, m_s = -1\rangle - \left(\sqrt{\frac{1}{12}} + \sqrt{\frac{1}{8}}\right)|s = 1, m_s = 0\rangle - \left(\sqrt{\frac{1}{12}} - \sqrt{\frac{1}{8}}\right)|s = 0, m_s = 0\rangle \\ &+ \sqrt{\frac{1}{12}}|s = 1, m_s = 1\rangle,\end{aligned} \tag{39}$$

where $s = s_1 + s_2$ is the total electronic spin. Therefore, the inter-atomic potential for the total-hyperfine-spin $F = 3/2$ scattering channel is given in terms of the electronic spin-singlet and spin-triplet counterparts $V_{\rm s}$ and $V_{\rm t}$ by

$$\begin{aligned}V_{3/2} =& \left[\frac{1}{2} + \left(\sqrt{\frac{1}{12}} + \sqrt{\frac{1}{8}}\right)^2 + \frac{1}{12}\right] V_{\rm t} + \left(\sqrt{\frac{1}{12}} - \sqrt{\frac{1}{8}}\right)^2 V_{\rm s} \\ =& \frac{(19 + 2\sqrt{6})V_{\rm t} + (5 - 2\sqrt{6})V_{\rm s}}{24} \\ \simeq& 0.996V_{\rm t} + 0.004V_{\rm s}.\end{aligned} \tag{40}$$

Similarly, we expand the hyperfine state $|F = \frac{1}{2}, m_F = \frac{1}{2}\rangle$ in the electronic-spin basis by using the Clebsch-Gordan coefficients. The resulting inter-atomic potential for the total-hyperfine-spin $F = 1/2$ scattering channel is given by

$$\begin{aligned}V_{1/2} =& \frac{(26 + 2\sqrt{6} + 2\sqrt{2})V_{\rm t} + (10 - 2\sqrt{6} - 2\sqrt{2})V_{\rm s}}{36} \\ \simeq& 0.94V_{\rm t} + 0.06V_{\rm s}.\end{aligned} \tag{41}$$

It is clear from Eqs. (40) and (41) that the atomic potential $V_{3/2}$ is almost equal to the spin-triplet potential V_t while there is an approximately 5% mixing of the spin-singlet potential V_s in $V_{1/2}$. However, it can be seen from the numerically calculated V_s and V_t for LiRb (see Figs. 1 and 2 in Ref. [2]) that the energy difference between two neighboring bound states of V_t is smaller than 5% of V_s. Therefore, we cannot make a precise evaluation of the scattering length $a_{1/2}$ without exactly solving the Schrodinger equation with potential $V_{1/2}$, which is beyond the scope of this work. Instead, using the values of $a_\mathrm{t} = 24\, a_\mathrm{B}$ and $a_\mathrm{s} = -64\, a_\mathrm{B}$ (a_B is the Bohr radius), which have been numerically calculated in Ref. [3], it is likely that the difference $a_{3/2} - a_{1/2}$ has the same order of magnitude as a_s and a_t. In the main text, we take $|a_{3/2} - a_{1/2}| = 50\, a_\mathrm{B}$ in our order-of-magnitude estimation of the spin current density.

SUPPLEMENTARY REFERENCES

[1] Haug, H. & Jauho, A.-P. *Quantum Kinetics in Transport and Optics of Semiconductors* (Springer, 2008).

[2] Korek, M., Allouche, A. R., Kobeissi, M., Chaalan, A., Dagher, M., Fakherddin, K. & Aubert-Frecon, M. Theoretical study of the electronic structure of the LiRb and NaRb molecules. *Chem. Phys.* **256**, 1–6 (2000).

[3] Ouerdane, H. & Jamieson, M. J. Scattering parameters for cold Li-Rb and Na-Rb collisions derived from variable phase theory. *Phys. Rev. A* **70**, 022712 (2004).

[4] Brink, D. M. & Satchler, G. R. *Angular Momentum* 3rd Ed. (Oxford University Press, 1994).
